# Estradiol enhances influenza vaccine responses through B cell metabolic reprogramming in female mice

**DOI:** 10.1128/mbio.03965-25

**Published:** 2026-02-26

**Authors:** Laura A. St Clair, Emily G. Watters, Anna Yin, Jennifer A. Liu, Sabal Chaulagain, Elizabeth A. Thompson, Sabra L. Klein

**Affiliations:** 1 W. Harry Feinstone Department of Molecular Microbiology and Immunology, Johns Hopkins University Bloomberg School of Public Health 25802, Baltimore, Maryland, USA; Duke University School of Medicine, Durham, North Carolina, USA

**Keywords:** influenza, estrogen, aging, neutralizing antibodies, immunometabolomics, sex difference

## Abstract

Reproductive-aged females mount stronger antibody responses to influenza vaccination than males, with the sex difference waning in older age. Estradiol has been implicated as a driver, but the mechanisms mediating how estradiol affects B cell function remain elusive. Adult (3 months) and aged (17 months) male and female mice were vaccinated and boosted with inactivated influenza vaccine. Metabolomics analysis of splenic B cells revealed that adult female B cells were enriched in lipid metabolic pathways, whereas B cells from males were enriched in central carbon-associated pathways following vaccination. B cells from vaccinated adult females exhibited greater expression of mTOR and related proteins than those from males, a difference diminished in aged mice. In adult females, estradiol depletion reduced, and replacement increased, mTOR activity in B cells, particularly in germinal center B cells and plasmablasts in lymphoid tissues, and plasma cells in bone marrow. In males, neither testosterone depletion nor repletion altered B cell metabolism. These findings are consistent with evidence that estradiol enhances mTOR activation via estrogen receptor α (ERα) signaling, suggesting coordinated regulation between estrogen and mTOR signaling in B cells. Inhibition of mTOR with rapamycin impaired vaccine-induced antibody responses and protection in adult females. In aged females, supplementation with estradiol or treatment with a selective ERα agonist increased mTOR signaling and enhanced antibody responses compared with mock-treated aged females. These data identify estrogen signaling as a regulator of B cell metabolism that supports greater expansion and function of antibody-secreting cells following vaccination in females compared with males.

**IMPORTANCE:**

Vaccine-induced immunity differs between the sexes, with adult females mounting stronger antibody responses to influenza vaccination than age-matched males. We show that estradiol in females regulates B cell metabolism to promote the maturation and metabolic activation of antibody-secreting B cells, thereby enhancing humoral immunity and protection following vaccination. mTOR signaling in B cells was greater in adult females than males after vaccination, which was diminished with aging or depletion of estradiol. Therapeutic treatment of aged females with either estradiol or a selective estrogen receptor α modulator increased mTOR signaling and improved vaccine-induced antibody responses, thereby eliminating the effects of aging on influenza immunity. Harnessing estrogen-signaling mechanisms to improve responses to influenza vaccines could be a novel therapeutic strategy to improve public health.

## INTRODUCTION

Adult females produce greater antibody responses than age-matched males following vaccination against either seasonal or pandemic influenza strains in humans and mice (1–6). Greater antibody responses in females are correlated with greater estradiol (E2) concentrations in humans (7). Mouse models demonstrate that E2 enhances immunity and protection induced by inactivated influenza vaccine (IIV). Removal of the ovaries and E2 secretion, via surgery or by the presence of testes in XX four core genotype (FCG) mice, reduces, whereas treatment of gonadectomized females with E2 or the presence of ovaries and E2 secretion in XX or XY FCG mice increases immunity and protection following vaccination (4–7). Sex differences in IIV-induced immunity wane with aging in both humans and mice, with the age-related decline being greater in aged females than males, which likely reflects cessation of ovarian function (7–11). These findings suggest that estrogen signaling plays a central role in shaping vaccine-induced humoral immune responses in females. The mechanism mediating how steroids like E2 cause increased antibody protection by B cells, however, remains incompletely defined.

B cells undergo metabolic reprogramming to meet the energetic and biosynthetic demands of activation, differentiation, and antibody production (12–25). There is increased nutrient uptake, engagement of anabolic pathways, and mitochondrial remodeling to sustain B cell proliferation and antibody synthesis (14–17). Sex differential regulation of metabolism is well documented in diverse organ systems. In cardiac and skeletal muscle and liver, male tissues preferentially utilize fatty acid oxidation, whereas females rely more on glycolysis in basal conditions with the patterns of cellular metabolism reversing between the sexes in activated states (26–34). Whether sex differential reciprocal metabolic reprogramming exists within the immune system, particularly in B cells, has not been reported and formed the basis of the current study. Furthermore, how steroids, including E2, alter metabolic activity in B cells to result in both sex and age-related differences in antibody secretion following vaccination was further explored.

## RESULTS

### Adult females have greater antibody responses and protection against influenza than adult males

**Fig 1:**
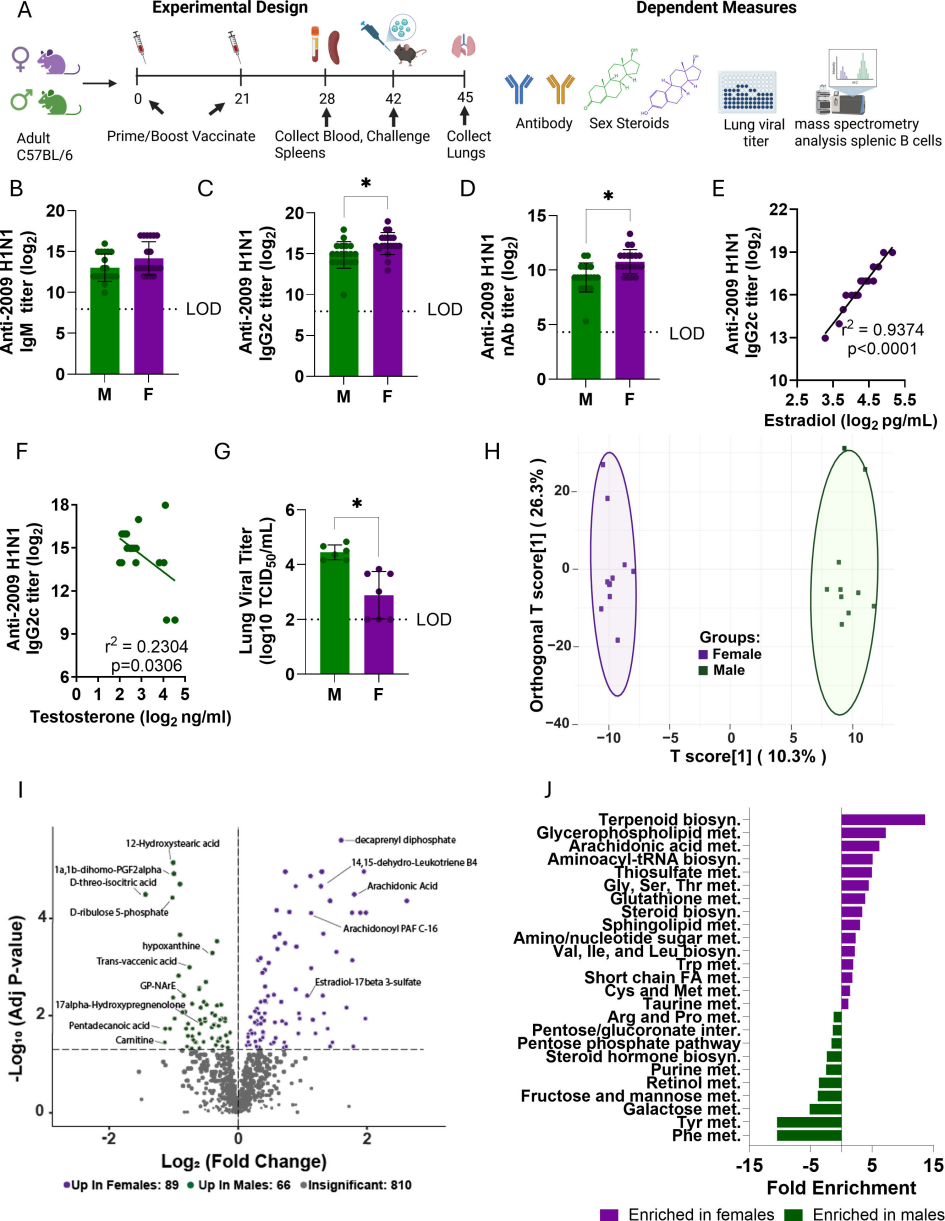
Females have greater inactivated influenza vaccine (IIV)-induced immunity and protection than males, corresponding with distinct B cell metabolic programs. (**A**) Experimental design (image generated using Biorender). Adult (3-month-old) male (green) and female (purple) C57BL/6 mice were primed and boosted with IIV or PBS (mock) at a 3-week interval, and tissues were collected at the indicated time points (*n* = 10/sex/group). Serum collected at 28 days post-vaccination (dpv) was used to measure (**B**) anti-H1N1 IgM, (**C**) anti-H1N1 IgG2c, (**D**) neutralizing antibody (nAb) titers, (**E**) estradiol (E2) in females, and (**F**) testosterone in males. (**G**) Lung homogenates were used to assess viral titers at 3 days post-challenge with an maA/Cal/09 H1N1 drift variant. (**H–J**) Splenic B cells isolated at 28 dpv from mock- and IIV-vaccinated males and females (*n* = 10/sex/group) were subjected to untargeted metabolomics analysis. All samples from vaccinated mice were normalized to sex-matched mock-vaccinated controls. (**H**) Score plot of OPLS-DA model in vaccinated animals; the model had strong model performance (R²Y = 0.991, *Q*² = 0.791) and was validated by 200-permutation testing (*P* < 0.005). (**I**) Volcano plot with the *x*-axis representing log₂ fold change (log₂FC) and the *y*-axis indicating –log₁₀ false discovery rate (FDR)-adjusted *P*-value (FDR < 0.05) of all identified metabolites in vaccinated animals. Purple and green dots represent metabolites significantly enriched in females and males, respectively. Labeled points highlight metabolites of interest. (**J**) Quantitative Metabolite Set Enrichment Analysis (QSEA) of significantly altered metabolites performed using MetaboAnalyst 5.0 with KEGG pathway annotation. Enrichment *P*-values were calculated using a hypergeometric test. Statistical analyses: (**B–D**, **G**) Student’s *t*-test; (**E, F**) simple linear regression; (**H**) OPLS-DA model with permutation testing (200 iterations); (**I**) FDR-adjusted *P*-values (*P* < 0.05) were calculated using unpaired two-sided *t*-tests with Benjamini-Hochberg correction; (**J**) hypergeometric test for pathway enrichment. *P < 0.05. Abbreviations: Arg = arginine, biosyn. = biosynthesis, Cys = cysteine, FA = fatty acid, Gly = glycine, Ile = isoleucine, inter. = interconversions, Leu = leucine, met. = metabolism, Met = methionine, Phe = phenylalanine, Pro = proline, Ser = serine, Thr = threonine, Trp = tryptophan, Tyr = tyrosine, Val = valine.

Consistent with previous studies (4–7), at 28 days post-vaccination (dpv) with inactivated 2009 H1N1 virus (IIV) (Fig. 1A), anti-2009 H1N1 IgM titers were comparable between sexes (Fig. 1B), but class-switched IgG2c and neutralizing antibody (nAb) titers were greater in females than males (Fig. 1C and D). Females also had greater IgG2c titers than males against a panel of antigenically drifted mouse-adapted 2009 H1N1 (maA/Cal/09 H1N1) viruses that contained mutations (M) in the immunodominant head of hemagglutinin (5) (Fig. S1A through D). In contrast, anti-2009 H1N1 IgG1 and IgG3, but not IgG2b, titers were greater in vaccinated males than females (Fig. S1E through G). Greater serum concentrations of E2 were positively associated with greater anti-2009 H1N1 IgG2c titers in females (Fig. 1E), whereas greater testosterone (T) concentrations in males were weakly, but significantly, associated with lower anti-2009 H1N1 IgG2c titers (Fig. 1F). Following challenge with a 1M drift variant of A/Cal/09 H1N1(5) at 42 dpv, vaccinated females had lower lung viral titers than males at 3 days post-challenge (Fig. 1G). These data suggest that sex differences in vaccine-induced immunity reflect both differences in antibody magnitude and in the IgG isotype distribution, which might be estrogen-dependent.

### Metabolomic profiling reveals divergent pathways in male and female B cells following influenza vaccination

To determine if greater neutralizing antibody responses in females were associated with metabolic changes in B cells, untargeted metabolomic profiling was conducted using total splenic B cells isolated at 28 dpv from mock- and IIV-vaccinated male and female mice. Multivariate modeling using orthogonal partial least squares discriminant analysis (OPLS-DA) (Fig. S1H) and differential abundance analysis (Fig. S1I) revealed distinct baseline metabolic profiles between male and female B cells in mock-vaccinated animals, indicating intrinsic sex differences in B cell metabolic programming. To isolate vaccine-induced metabolic changes, metabolic responses in B cells from vaccinated mice were expressed as within-sex log_2_ fold changes relative to sex-matched mock-vaccinated mice, controlling for baseline differences. After baseline adjustment, OPLS-DA demonstrated that metabolic profiles of B cells from vaccinated females and males remained distinct (Fig. 1H).

Quantitative Metabolite Set Enrichment Analysis (QSEA), performed in MetaboAnalyst using the Kyoto Encyclopedia of Genes and Genomes (KEGG) database, further revealed enrichment of central carbon metabolic pathways in female B cells and lipid-associated pathways in male B cells at baseline (Fig. S1J). Analysis of normalized post-vaccination data identified 155 metabolites that differed significantly between vaccinated females and males (log_2_ fold change [FC] > 1, false discovery rate [FDR]-adjusted *P* < 0.05), with 89 enriched in B cells from females and 66 enriched in B cells from males (Fig. 1I). QSEA of vaccinated samples revealed enrichment of lipid-associated pathways in female B cells and central carbon metabolism in male B cells following vaccination (Fig. 1J). Although terpenoid biosynthesis ranked as the top enriched pathway, this enrichment was driven by a single lipid intermediate, so further analyses focused on the broader enrichment of lipid pathways in females and central carbon metabolism in males. These findings indicate that vaccination elicits sex differential engagement of distinct metabolic pathways in B cells.

**Fig 2:**
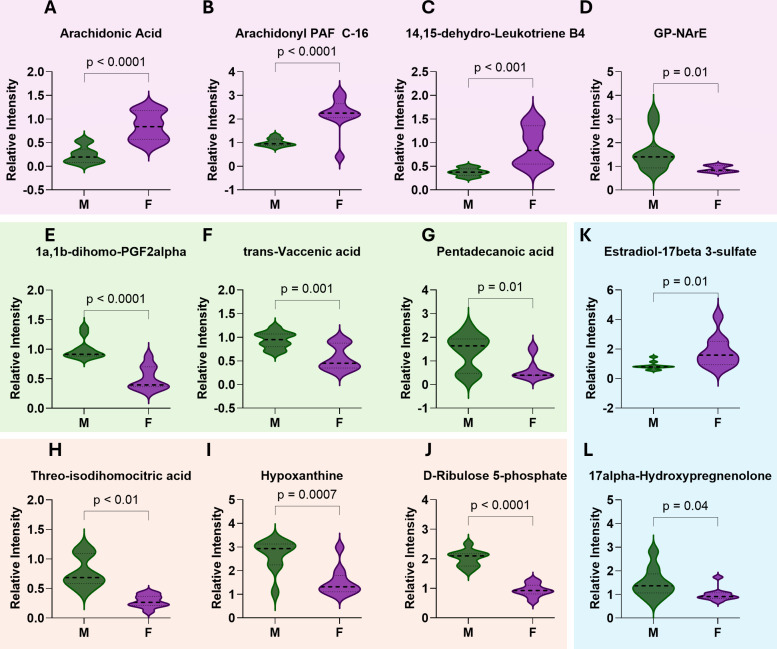
Vaccination induces sex-differential metabolic programming of B cells across lipid, central carbon, and steroid pathways. (**A–D**, pink panel) Arachidonic acid and derivatives, enriched in females (**A–C**) or in males (**D**) (*N* = 10/sex). (**E–G**, green panel) Bioactive fatty acids enriched in males. (**H–J**, orange panel) Central carbon metabolism intermediates, including tricarboxylic acid (TCA) cycle, purine metabolism, and pentose phosphate pathway, enriched in males. (**K, L**, blue panel) Steroid metabolites enriched in either females or males. Violin plots show data distribution and median (dashed line). Statistical comparisons were performed using multiple unpaired *t*-tests, and adjusted *P*-values were calculated using the two-stage step-up method of Benjamini, Krieger, and Yekutieli to control the FDR. Values represent metabolite intensities normalized to same-sex mock controls.

To further define sex differential metabolic activity in B cells following vaccination, we focused on the top 50 significantly different metabolites (Fig. 2; Fig. S2 and S3). Because multiple, overlapping lipid pathways were significantly enriched in females, we analyzed lipid-associated metabolites collectively rather than individual pathways. Within these lipid networks, B cells from females showed enrichment for arachidonic acid (Fig. 2A) and its derivatives, including arachidonoyl PAF C-16 and 14,15-dehydro-leukotriene B4 (Fig. 2B–C), which can promote B cell activation in part through PI3K/Akt/mTOR signaling (35–38). In contrast, the arachidonic acid derivative glycerophospho-N-arachidonoyl ethanolamine (GP-NArE, Fig. 2D) was enriched in males and is a precursor of molecules that inhibit lymphocyte proliferation and induce DNA damage (39, 40). Females also had greater abundance of long-chain fatty acids (LCFAs), whereas males showed higher levels of very long-chain fatty acids (VLCFAs; Fig. S2 and Table S1). Because VLCFAs are oxidized less efficiently by mitochondria (41, 42), these findings suggest sex differences in lipid utilization. Consistent with this interpretation, B cells from females had lower levels of free carnitine compared to males (Fig. S2A), which may reflect increased carnitine consumption for mitochondrial import via carnitine palmitoyl transferase 1A (CPT1A), although acyl-carnitine species were not directly measured in this data set.

B cells from males were enriched in bioactive fatty acids, such as 1a,1b-dihomo-PGF2α (Fig. 2E), an anti-inflammatory eicosanoid (43), trans-vaccenic acid (Fig. 2F), and pentadecanoic acid (Fig. 2G), which have been implicated in inhibition of Akt phosphorylation and activation of AMPK, respectively (44–46). B cells from males also exhibited elevated levels of intermediates in the tricarboxylic acid (TCA) cycle (Fig. 2H; Fig. S3A through E), purine metabolism (Fig. 2I; Fig. S3F), and the pentose phosphate pathway (Fig. 2J; Fig. S3G and H), indicating greater reliance on central carbon metabolism. Finally, steroid metabolites showed sex differential signatures, with levels of estradiol-17β 3-sulfate, a storage form of E2, being higher in B cells from females (Fig. 2K), whereas 17α-hydroxypregnenolone, a precursor in testosterone biosynthesis, was higher in B cells from males (Fig. 2L).

### B cells undergo sex differential shifts in mTOR pathway and CPT1A protein expression following influenza vaccination

**Fig 3:**
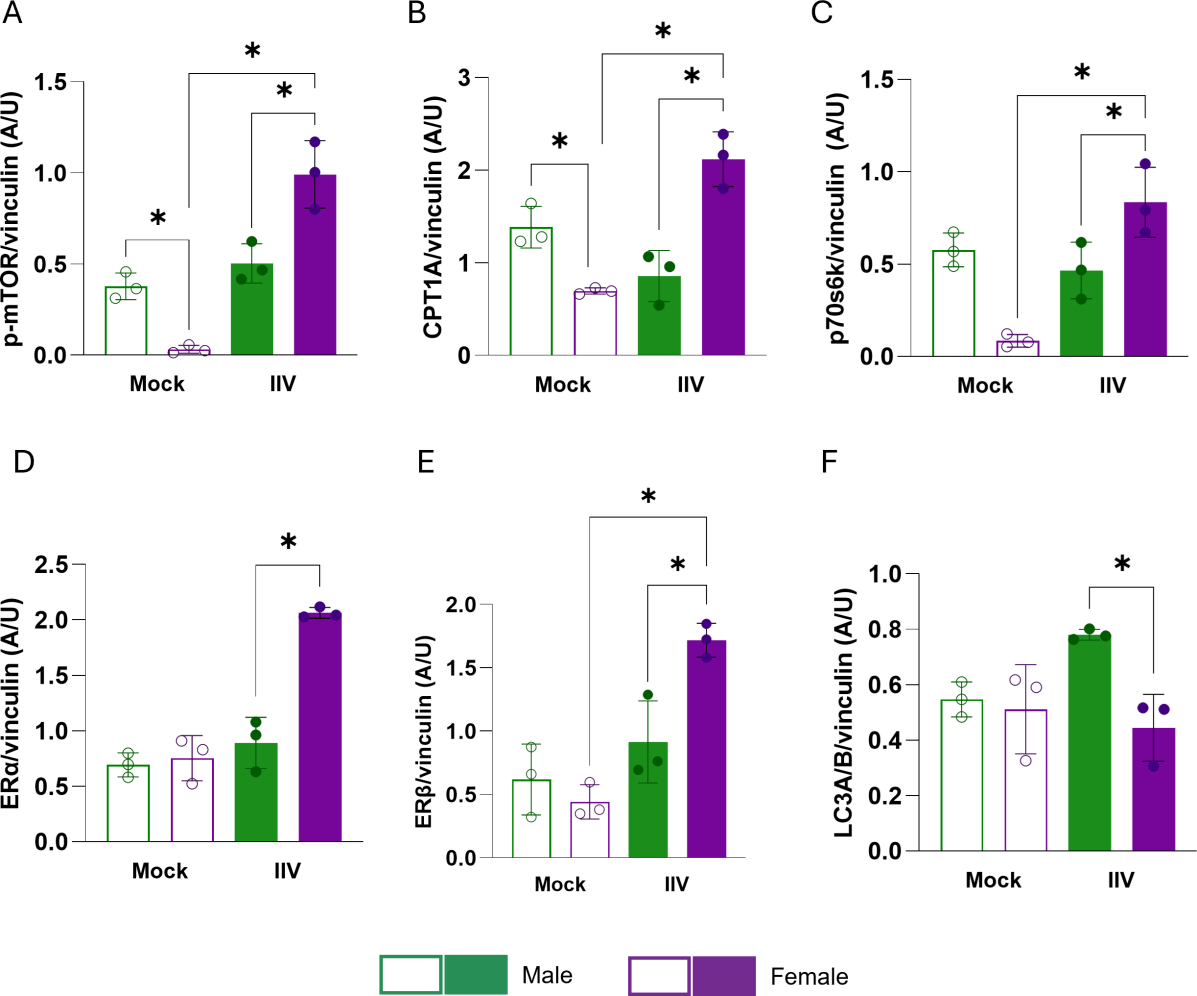
Vaccination induces a shift in mTOR signaling and fatty acid oxidation in splenic B cells from females but not males. Adult male and female mice were vaccinated and boosted as in Fig. 1 (*n* = 3/sex/group). At 28 days post-vaccination, splenic B cells were isolated, and whole-cell protein lysates were analyzed by Western blot. (**A–F**) Densitometric quantification of p-mTOR (**A**), CPT1a (**B**), p-p70S6K (**C**), ERα (**D**), ERβ (**E**), and LC3A/B (**F**) was normalized to vinculin (loading control). Each point represents an individual mouse. Bars indicate mean ± SEM. Statistical significance was determined by one-way ANOVA with Tukey’s multiple comparisons test, *P < 0.05.

Because the metabolomics data revealed elevated mTOR-activating lipids in females (e.g., arachidonic acid and derivatives) (35–38) and mTOR-suppressive lipids in males (e.g., trans-vaccenic acid and pentadecanoic acid) (44–46), we hypothesized that there might be sex differences in the activation of mTOR signaling in B cells following vaccination. To test this, splenic B cells were isolated at 28 dpv from mock- and IIV-vaccinated adult male and female mice, and lysates were analyzed for the expression of phosphorylated mTOR (p-mTOR) and related signaling proteins (Fig. 3; Fig. S4). In mock-vaccinated mice, male B cells had greater expression of p-mTOR (Fig. 3A; Fig. S4A); CPT1A, the rate-limiting enzyme of fatty acid oxidation (Fig. 3B; Fig. S4B); and phosphorylated p70S6K (p-p70S6K), a downstream mTOR target (Fig. 3C; Fig. S4C), compared with females. After vaccination, B cells from females showed greater p-mTOR, CPT1A, and p-p70S6K expression than those from males, indicating selective activation of mTOR signaling and lipid metabolism pathways in females. In contrast, in B cells from males, IIV did not alter these proteins as compared with mock vaccination. B cells from vaccinated females also showed increased expression of estrogen receptor alpha (ERα) and estrogen receptor beta (ERβ) relative to cells from either males or mock-vaccinated females (Fig. 3D and E; Fig. S2D and E), suggesting that E2 signaling was upregulated in B cells from females following vaccination. Finally, expression of LC3A/B, a marker of autophagy and negative regulator of mTOR signaling, was uniquely increased by vaccination in B cells from males (Fig. 3F; Fig. S4F).

To assess the ubiquity of vaccine-induced mTOR activation in B cells from females, mice were vaccinated and boosted with inactivated A/Hong Kong/68/H3N2 vaccine prior to sample collection (Fig. S5). As observed with 2009 H1N1, B cells from H3N2 vaccinated females showed elevated p-mTOR, CPT1A, and p-p70S6K expression, while B cells from vaccinated males showed greater expression of LC3A/B (Fig. S5). Together, these data highlight that vaccination induces activation of mTOR signaling and fatty acid oxidation in B cells from females, while B cells from males seem to rely on alternative pathways, such as glycolysis and central carbon metabolism.

### mTOR signaling is required for vaccine-induced class switching and protection in females

**Fig 4:**
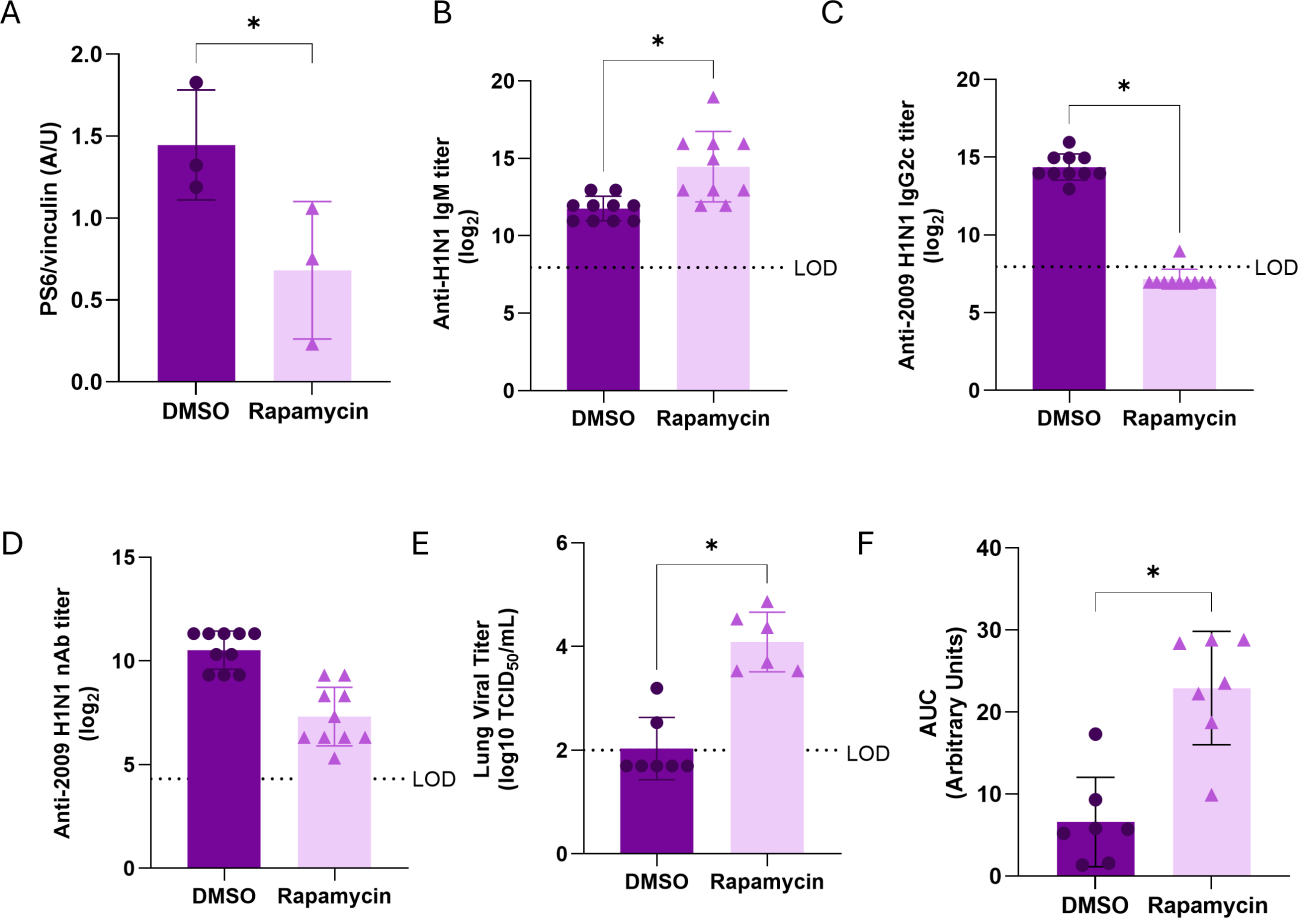
Inhibition of mTOR signaling impairs vaccine-induced antibody responses and protection in female mice. Adult female mice were treated daily by intraperitoneal (i.p.) injection with either vehicle or 8 mg/kg rapamycin for 21 days and were vaccinated and boosted as described in Fig. 1 (*n* = 10/treatment). At 28 days post-vaccination (dpv), a subset of mice (*n* = 3/treatment) were euthanized to measure protein expression of p-p70S6K (**A**); in total splenic B cells, all remaining mice were also bled at 28 dpv for analysis of anti-H1N1 IgM (**B**), anti-H1N1 IgG2c (**C**), and nAb (**D**) titers. Mice were challenged at 42 dpv, and morbidity was assessed via analysis of lung viral titers at 3 days post-challenge (**E**) and change in body mass which is displayed as absolute area under the curve (AUC; **F**). Each point represents an individual mouse. Bars indicate mean ± SEM. Statistical significance was determined by Student’s *t-*test, *P < 0.05.

To test whether mTOR signaling was required for enhanced vaccine-induced antibody responses and protection in females, vaccinated female mice were treated daily with either the mTOR antagonist rapamycin or vehicle during the vaccination period (0–21 dpv). At 28 dpv, rapamycin reduced p-p70S6K expression in B cells (Fig. 4A; Fig. S6A). Rapamycin-treated females exhibited greater anti-2009 H1N1 IgM (Fig. 4B) and lower anti-2009 H1N1 IgG2c (Fig. 4C) and nAb (Fig. 4D) titers compared to vehicle-treated controls, consistent with impaired class-switch recombination. Following challenge with the 1M drift variant of 2009 H1N1 at 42 dpv, lung viral titers were greater in rapamycin-treated than vehicle-treated vaccinated females (Fig. 4E), indicating reduced viral clearance. Rapamycin-treated vaccinated females also experienced greater morbidity, measured by body mass loss over a 3-day period after infection, than vehicle-treated vaccinated females (Fig. 4F). Although these data indicate that mTOR signaling in B cells contributes to vaccine-induced immunity, rapamycin suppressed mTOR signaling systemically, so effects in B cells may reflect both direct and indirect effects (e.g., CD4+ T cells) on B cells.

To determine whether activating mTOR could improve vaccine responses in males, males received daily injections of the mTOR agonist MHY1485, the only direct agonist commercially available, during their vaccination. At the recommended dose, MHY1485 failed to enhance mTOR signaling in B cells from males (Fig. S6A and B) and did not alter antibody responses (Fig. S6C through E) or protection (Fig. S6F through G). The limited *in vivo* efficacy of MHY1485 precludes drawing conclusions and highlights that better agonists are needed for research and therapeutic use.

### Estradiol promotes expansion of B cell subsets associated with antibody responses after influenza vaccination

**Fig 5:**
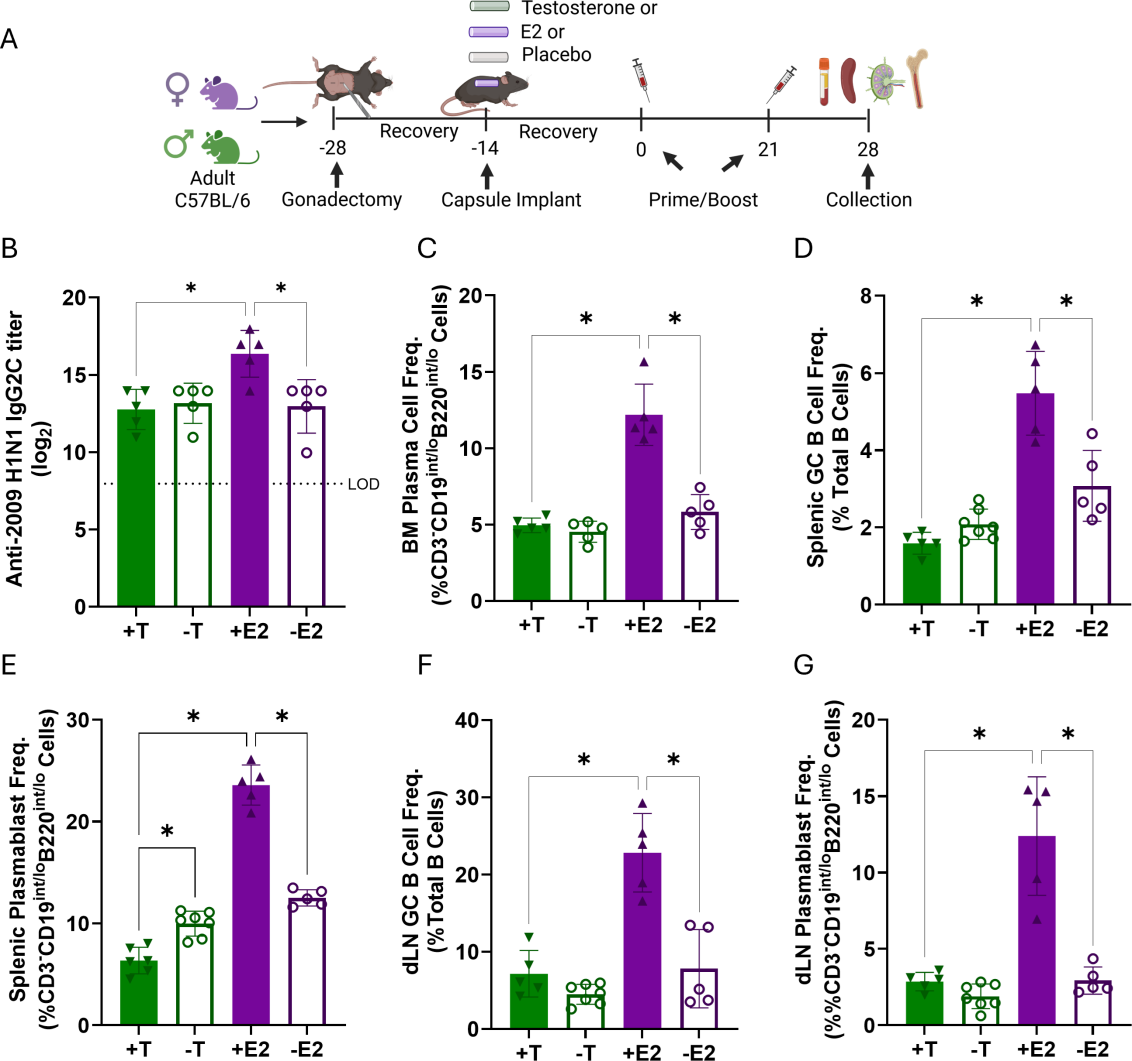
Estradiol promotes expansion and differentiation of antibody-producing B cell subsets following vaccination. (**A**) Experimental design: adult (3 months) male and female mice (*N* = 5/sex/group) were gonadectomized and implanted with placebo capsules or capsules containing estradiol (E2, females) or testosterone (T, males) and then vaccinated (image generated using Biorender). At 28 days post-vaccination (dpv), serum and lymphoid tissues were collected. (**B**) anti-H1N1 IgG2c responses were measured at 28 dpv. Multi-parameter spectral flow cytometry was used to analyze frequencies of bone-marrow plasma cells (**C**), splenic germinal center (GC) B cells and plasmablasts (**D, E**), and draining lymph node (dLN) GC B cells and plasmablasts (**F–G**). Statistical significance was determined by one-way ANOVA with Tukey’s multiple comparisons test, *P < 0.05.

To determine whether sex steroid signaling regulates B cell metabolic activation, and whether these effects are restricted to specific B cell subsets, we gonadectomized male and female mice and replaced T in males and E2 in females, with subsets of gonadectomized males (-T) and females (-E2) receiving placebo (Fig. 5A). As a bioassay of hormone replacement efficacy, wet weights of reproductive tissues were recorded, with E2 treatment in females and T treatment in males increasing the weights of the uterine horns and seminal vesicle, respectively, as compared with placebo controls (Fig. S7A). While manipulation of T in males had no effect on anti-2009 H1N1 IgG2c titers, E2 treatment in females increased anti-2009 H1N1 IgG2c titers, with gonadectomy in the absence of E2 treatment resulting in anti-2009 H1N1 IgG2c titers that were consistent with those of males (Fig. 5B). In contrast, gonadectomized males receiving T had higher titers of anti-2009 H1N1 IgG1, IgG2b, and IgG3 titers than E2-treated females. Gonadectomized females without hormone replacement had greater anti-2009 H1N1 IgG1, IgG2b, and IgG3 titers than females receiving E2 (Fig. S7B through D). These data suggest that gonadal steroids influence IgG class switching and subclass distribution following vaccination.

Flow cytometric analysis of B cell subsets, including B-1 B cells, transitional B cells (T1, T2, and T3), marginal zone (MZ), follicular (FO), germinal center (GC) B cells, and plasmablasts in spleens and draining lymph nodes (dLN; Fig. S8) and plasma cells in the bone marrow (BM), was performed 28 dpv. Among vaccinated females, the presence of E2 expanded populations of plasma cells in the bone marrow (Fig. 5C) and antibody-secreting cells (ASCs) and germinal center B cells in spleens and dLNs (Fig. 5C through G) as compared with either females depleted of E2 or males. In contrast, the presence of E2 reduced frequencies of FO B cells in the spleen and dLN (Fig. S7E and F), coinciding with reduced transitional B cells (T1-3) in spleen and dLNs and reduced immature B cells in BM (Fig. S7G through M). Frequencies of innate-like B cells (B-1 and marginal zone subsets) also varied across groups, but the patterns were inconsistent and less clearly associated with hormone treatment (Fig. S7N through Q). In contrast, depletion of T in males increased frequencies of transitional B cells (T1-3) in lymphoid tissues (Fig. S7G through M). Together, these data suggest the presence of E2 supports increased B cell activation and differentiation and reduces the presence of more immature phenotypes. Because gonadal steroids altered B cell frequencies following vaccination, particularly increasing frequencies of ASCs in the presence of E2 in females, we next sought to determine if gonadal steroids also regulated metabolic activity within B cell subsets.

### Estradiol enhances metabolic remodeling across B cell subsets in females after influenza vaccination

**Fig 6:**
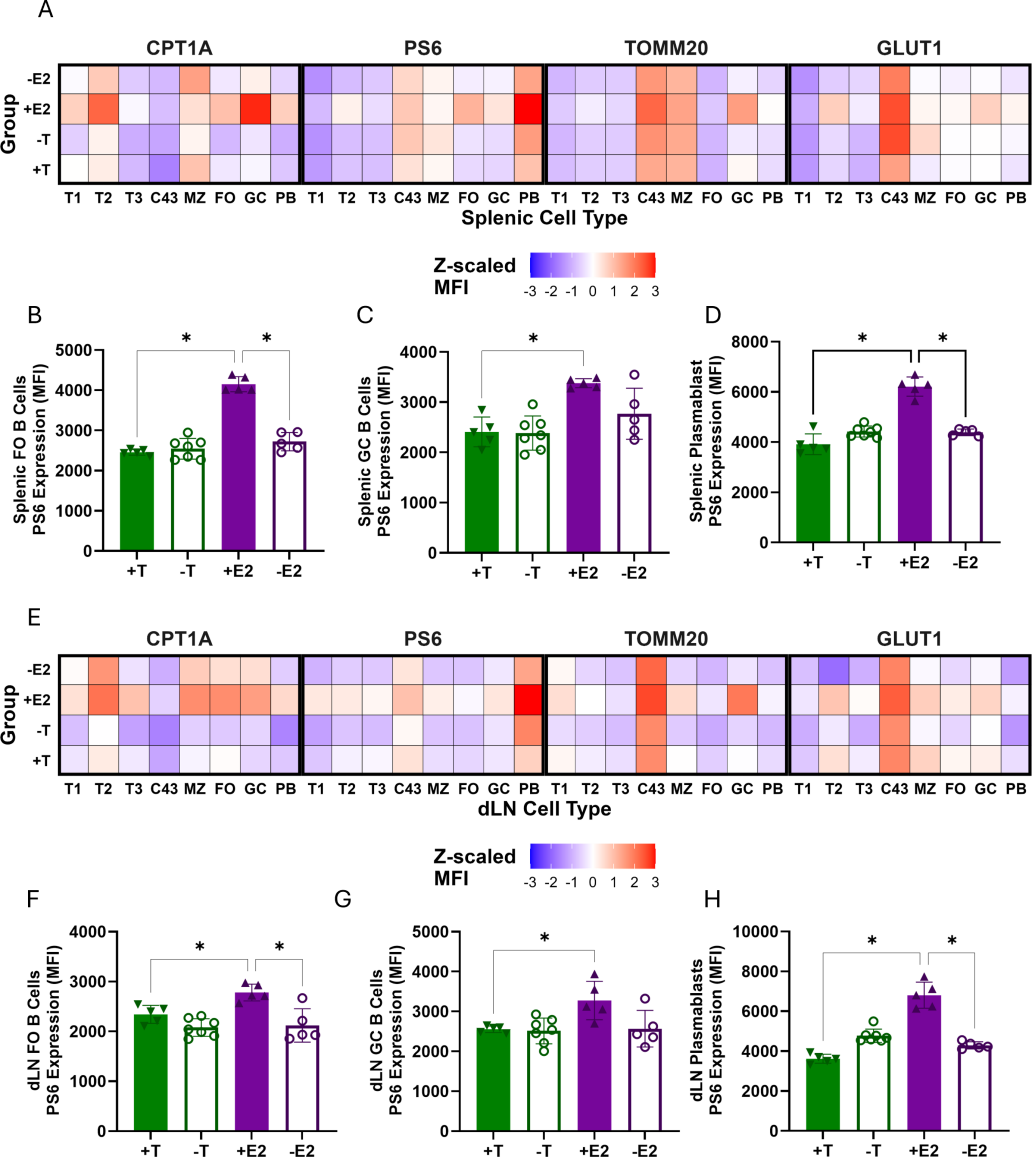
Estradiol regulates metabolic activity in antibody-secreting cells following vaccination. (**A**) Heatmap of *Z*-score–transformed mean fluorescence intensity (MFI) values for pS6, CPT1a, TOMM20, and GLUT1 in splenic B cell subsets at 28 days post-vaccination, generated in R using ggplot2. (**B–D**) Bar plots of pS6 expression (MFI) in follicular (FO), germinal center (GC), and plasmablast subsets from spleen. (**E**) Heatmap of *Z*-score–transformed MFI values for metabolic proteins across dLN B cell subsets. (**F–H**) Bar plots of pS6 expression (MFI) in FO, GC, and plasmablast subsets from dLN. Statistical analysis: one-way ANOVA with Tukey’s multiple comparisons, *P < 0.05.

Using flow cytometry, the expression of intracellular metabolic proteins including pS6 (mTOR signaling), CPT1a (fatty acid oxidation), TOMM20 (mitochondrial mass), and GLUT1 (glucose transport)(47) was analyzed in B cell subsets in lymphoid tissues and BM 28 dpv. In both the spleen and dLN, the presence of E2 consistently induced greater expression of multiple metabolic proteins within plasmablasts, FO, and GC B cells (Fig. 6). The addition of E2 increased pS6 expression in FO and GC B cells and plasmablasts (Fig. 6A through H), suggesting that E2 enhances mTOR signaling across the FO-GC-plasmablast axis rather than merely increasing the frequency of metabolically active plasmablasts. Although ps6 expression was greatest in plasmablasts across all groups (Fig. 6A), E2 treatment further elevated pS6 levels within plasmablasts from E2-treated females as compared with either males or females depleted of E2 (Fig. 6A and D), supporting a direct role for E2 in upregulating mTOR activity in ASCs. CPT1a also was elevated in FO B cells, GC B cells, and plasmablasts in lymphoid tissues and plasma cells in BM from females treated with E2 (Fig. S9), with increased expression also in T2 transitional B cells (Fig. 6A; Table S2). TOMM20, but not GLUT1, was elevated in GC B cells and plasmablasts from E2-treated vaccinated females, suggesting greater mitochondrial capacity within these subsets following vaccination (Fig. 6A and E; Fig. S9). These data highlight that the presence of E2 enhances metabolic activity in differentiating and antibody-producing B cell subsets in lymphoid tissues and BM. In contrast, there were only marginal effects of T in males on the expression of metabolic markers in B cells following vaccination.

### Estrogen signaling increases mTOR activity and improves vaccine responses in aged females

**Fig 7:**
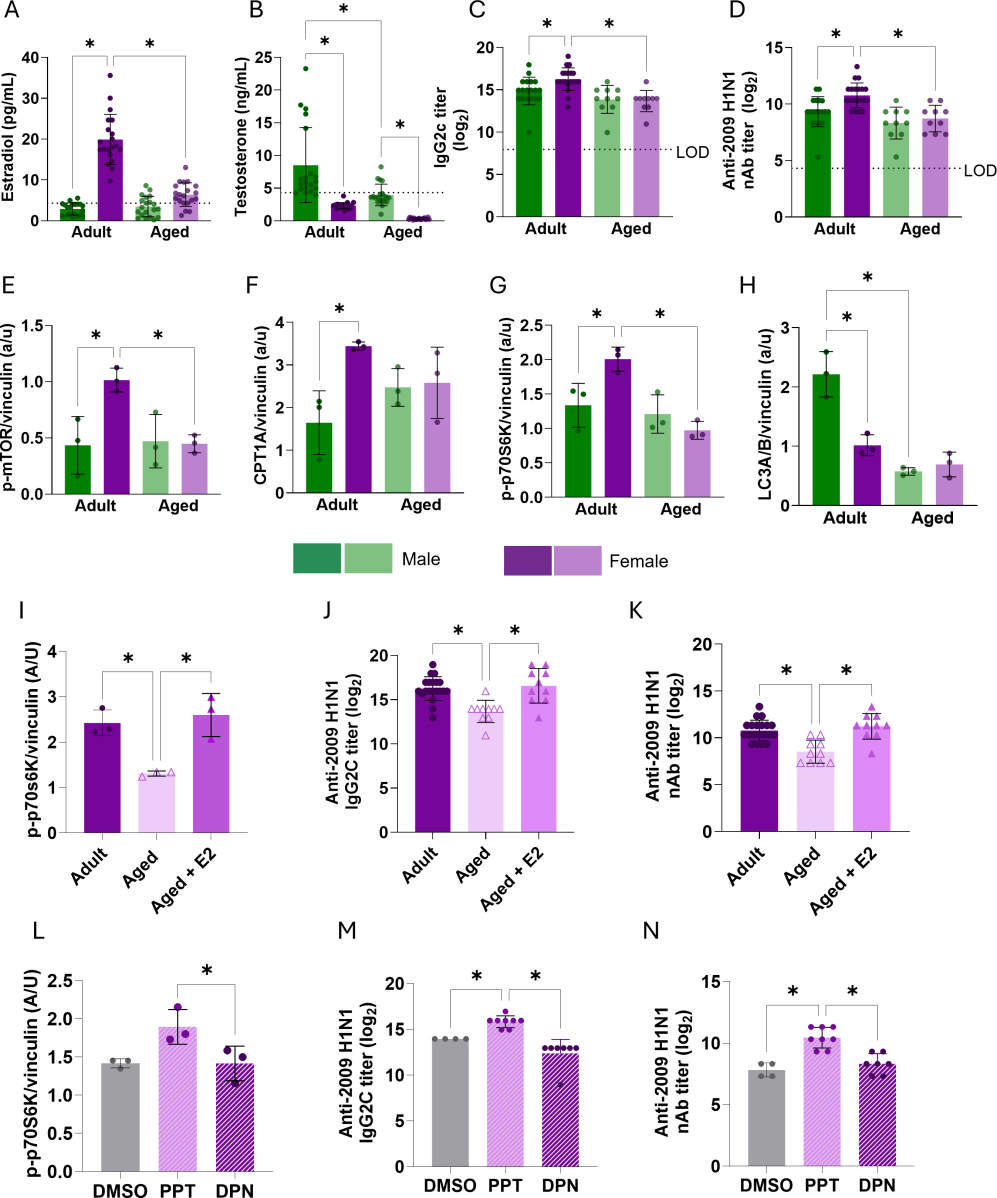
Estradiol and ERα signaling increases mTOR pathway protein expression and vaccine-induced antibody responses in aged females. (**A, B**) Serum concentrations of estradiol (E2) and testosterone (T) in adult and aged vaccinated male and female mice (*N* = 10–15/sex/age). (**C, D**) Serum anti-H1N1 IgG2c titers and neutralizing antibody (nAb) responses at 28 days post-vaccination in adult and aged mice. (**E–H**) Expression of p-mTOR, CPT1a, p-p70S6K, and LC3A/B in total splenic B cells from adult and aged males and females (*N* = 3/sex/age). (**I**) p-p70S6K expression in splenic B cells from vaccinated adult, placebo-treated aged, and E2-treated aged females (*N* = 3/group). (**J, K**) Anti-H1N1 IgG2c titers and nAb responses at 28 dpv in adult, placebo-treated aged, and E2-treated aged females (*N* = 10/group). (**L**) p70S6K expression in splenic B cells following treatment with vehicle, PPT (ERα agonist), or DPN (ERβ agonist) (*N* = 3/group). (**M, N**) Anti-H1N1 IgG2c titers and nAb responses at 28 dpv in vehicle-, PPT-, and DPN-treated aged females (*N* = 4-8/group). Statistical analysis: one-way ANOVA with Tukey’s multiple comparisons, *P < 0.05.

Age-related reductions in gonadal steroids, particularly E2, provide another system for examining the immunometabolic effects of depletion and repletion of E2 on vaccine-induced immunity. Serum concentrations of E2 in females and T in males were reduced in aged (17 months) compared with adult (3 months) mice, with a more pronounced age-related reduction in E2 (Fig. 7A and B). At 28 dpv, anti-2009 H1N1 IgG2c (Fig. 7C) and nAb (Fig. 7D) titers were significantly lower in aged compared with adult females, whereas aged males showed a downward trend that did not reach statistical significance. These data are consistent with our prior observations (6, 7) and suggest a greater sensitivity of female antibody responses to aging. In splenic B cells isolated at 28 dpv, the greater expression of p-mTOR, CPT1a, and p-p70S6K in adult females compared with adult males was mitigated in aged mice in which sex differences were not observed (Fig. 7E through G, Fig. S10A through C). There was an age-related reduction in the expression of p-mTOR and p70S6K, but not CPT1a, among females but not males (Fig. 7E through G; Fig. S10A through C). LC3A/B expression was greater in B cells from adult males than females, with no sex differences observed in B cells from aged mice (Fig. 7H; Fig. S10D).

To determine if E2 treatment of aged females could increase mTOR activity in B cells and vaccine-induced immunity, aged females were implanted with either placebo or E2 capsules and compared with adult females. At 28 dpv, splenic B cells from E2-treated aged females had levels of p-p70S6K expression that were comparable to levels in B cells from adult females and greater than expression levels in placebo-treated aged females (Fig. 7I; Fig. S10E). E2 treatment of aged females also increased anti-2009 H1N1 IgG2c (Fig. 7J) and nAb (Fig. 7K) titers to levels that were comparable with adult females and greater than titers in placebo-treated aged females.

Estradiol primarily signals through ERα or ERβ in B cells (48). To determine which receptor mediated the protective effects of E2 in aged females, females were treated with the ERα agonist propylpyrazole triol (PPT), the ERβ agonist diarylpropionitrile (DPN), or vehicle (DMSO) during the vaccination window. Treatment with PPT (122.2 ± 5.6 mg) but not DPN (70.67 ± 11.0 mg) increased uterine horn mass as compared with vehicle (73.75 ± 8.1 mg) treatment of aged females. Treatment with PPT, but not DPN, resulted in greater p-p70S6K expression in splenic B cells (Fig. 7L; Fig. S10F) as well as serum titers of anti-2009 IgG2c (Fig. 7M) and nAb (Fig. 7N) at 28 dpv as compared with vehicle treatment. In summary, E2 signaling through ERα therapeutically improves immunometabolic B cell activity in response to influenza vaccination in aged female mice.

## DISCUSSION

Greater immunity and protection from influenza vaccination in reproductive-aged females compared with males reflect the immunometabolic effects of E2 signaling through ERα in B cells. This mechanism explains how males and aged females have reduced class-switched protective antibodies than adult females, as previously reported (4–6). Antibody responses were measured as 2009 H1N1-specific titers following vaccination, rather than total circulating immunoglobulin levels, which also are sensitive to estrogenic effects (49). The metabolic effects of E2 involved activation of mTOR and lipid metabolism in B cells, particularly in plasma cells, plasmablasts, and GC B cells following vaccination. The therapeutic use of estrogen signaling to metabolically reprogram B cells and improve influenza vaccine-induced immunity and protection highlights the public health significance of these findings.

In response to 2009 H1N1, IgG subclass distribution differed by sex and was mediated by gonadal steroids. In gonadally intact mice, females mounted higher anti-2009 H1N1 IgG2c responses than males, whereas males exhibited higher anti-2009 H1N1 IgG1 and IgG3 titers. Estradiol replacement in gonadectomized females enhanced anti-2009 H1N1 IgG2c responses, whereas testosterone replacement in gonadectomized males increased anti-2009 H1N1 IgG1, IgG2b, and IgG3 titers. Together, these data indicate that gonadal steroids regulate IgG class switch recombination and subclass selection, consistent with prior reports (50, 51). In mice, IgG2c is functionally analogous to human IgG1, as both subclasses efficiently engage Fcγ receptors and mediate antiviral effector functions (52, 53). In contrast, murine IgG1 shares features with human IgG4 and is generally less effective at Fc-mediated viral clearance (54). While murine IgG3 and IgG2b share features with human IgG1 and IgG3 and can contribute to viral neutralization, these subclasses are often associated with Fc-dependent engagement of innate immune effector cells (55). Thus, the E2-dependent bias toward IgG2c observed in females suggests a qualitative skewing of humoral responses toward IgG subclasses with greater potential for antiviral effector activity.

Metabolomics revealed that following vaccination, B cells from adult males and females engage different metabolic pathways. In adult females, arachidonic acid and its derivatives have been reported to promote B cell activation and antibody production through PI3K/Akt signaling (35–38), and enrichment of LCFAs together with reduced free carnitine suggests increased flux into fatty acid oxidation (56). This aligns with higher CPT1A expression observed in B cells from adult females. In adult males, B cells showed enrichment of GP-NArE, a precursor of molecules reported to inhibit lymphocyte proliferation and induce DNA damage (39, 40). B cells from vaccinated males also contained levels of VLCFAs that are less efficiently β-oxidized. The presence of bioactive fatty acids such as 1a,1b-dihomo-PGF2α, trans-vaccenic acid, and pentadecanoic acid, which are implicated in inhibition of Akt phosphorylation or activation of AMPK (43–46), further supports antagonism of mTOR signaling in males. B cells from adult males exhibited greater LC3A/B expression, reflecting increased autophagy, which is negatively regulated by mTOR (57). These findings emphasize that vaccination drives B cells from females, including in the presence of E2, toward lipid metabolism coupled to mTOR signaling, while B cells from males shift toward central carbon metabolism and diminished mTOR engagement in response to vaccination.

These sex differential metabolic trajectories likely have direct functional consequences. Glycolysis provides rapid ATP to support short-term effector responses but is less suited for sustained activity (58). In contrast, lipid metabolism fuels mitochondrial respiration, supports biosynthesis, and sustains long-lived antibody-producing B cells (58–62). The shift toward central carbon metabolism observed in B cells from adult males after vaccination may enable brief activation but risks premature resource exhaustion, contributing to weaker and less durable antibody responses. In adult females, engagement of lipid metabolism and mTOR signaling appears to sustain GC proliferation, plasma cell differentiation, and long-lived antibody production (59–62). Thus, the divergent metabolic states of B cells from adult males and females help explain sex differences in vaccine-induced immunity. The gonadal steroid milieu likely influences the divergence of these metabolic states in B cells.

Depletion of E2 reduced and replacement increased the frequency and metabolic activity of GC B cells, plasmablasts, and plasma cells following vaccination in females. Testosterone, in contrast, had little effect on B cell metabolism or antibody responses, highlighting that sex steroids do not act interchangeably in regulating B cell function. The reduction in circulating E2 with age coincided with reduced expression of mTOR pathway proteins and antibody responses after vaccination. Therapeutic use of E2 or the selective estrogen receptor modulator (SERM) PPT in aged females restored B cell metabolic activity to levels consistent with adult females. We also observed that vaccination increased ERα and ERβ protein expression in B cells from females compared with males, suggesting that upregulation of ERs may underlie increased mTOR signaling lipid metabolism in adult female mice. ERα and mTOR are reported to participate in a positive feedback loop (63, 64), suggesting that E2 amplifies vaccine-induced B cell responses by reinforcing mTOR pathway activation, primarily through ERα.

There are limitations to this study, including the use of only mice, without confirmed translation in humans. Our study was performed in influenza-naïve mice, which does not capture the pre-existing immunity that impacts human responses to influenza vaccines (65). An additional consideration is how these findings may translate to pediatric vaccine responses. Vaccine-induced immunity in children prior to puberty reflects responses in individuals undergoing immune maturation in the context of low circulating gonadal steroids (66–69). Accordingly, the E2-mTOR axis defined here is unlikely to be a primary regulator of vaccine-induced antibody responses prior to puberty. Instead, our findings suggest that endocrine regulation of B cell immunometabolism may emerge after puberty and contribute to sex differences in antibody quality and protection among reproductive-aged adults. Determining how immunometabolic pathways function in early life is necessary for understanding sex differences in vaccine-induced immunity across the life course. Finally, the precise mechanisms associated with the bidirectional communication between ERα and mTOR in B cells following vaccination remain speculative. This study still represents an important advancement in our understanding of estrogen regulation of B cell metabolism as a central mechanism contributing to sex and age differences in vaccine-induced immunity. By linking endocrine signals to mTOR activity, lipid utilization, and the expansion of antibody-secreting B cells, this work highlights metabolism as a tractable axis through which humoral immunity can be shaped.

## MATERIALS AND METHODS

### Mice

Male and female C57BL/6CR mice (5–6 weeks old) were purchased from Charles River Laboratories (Frederick, MD, USA) and aged in-house to 8–10 weeks before experimentation. In-house aging enabled group housing of males before the onset of puberty, minimizing aggression and promoting social stability in laboratory mice (70). Aged (17-month-old) C57BL/6CR mice were provided by the National Institute on Aging aged rodent colony (Charles River origin). Mice were housed five per cage under biosafety level 2 conditions in the Johns Hopkins Bloomberg School of Public Health animal facility. Rooms were maintained at 20°C–22°C and 40°C–60% humidity, under a 12-hour light/dark cycle, with *ad libitum* access to food and water.

### Vaccination, challenge, and morbidity measurement

Mice were vaccinated intramuscularly in the right thigh with 10 µg of inactivated, mouse-adapted A/California/04/2009 H1N1 (ma2009 H1N1) vaccine, using a prime/boost strategy on a 3-week interval (4–7). For challenge studies, vaccinated mice were anesthetized with ketamine-xylazine (80 and 5 mg/kg, respectively) and infected intranasally with 10⁵ TCID₅₀ of a drift variant of ma2009 H1N1(5). Morbidity was assessed by daily monitoring of body mass and rectal temperature relative to baseline and by measuring lung viral titers at 3 days post-challenge.

### Surgical procedures

Adult mice (8–10 weeks old) underwent bilateral gonadectomy under ketamine-xylazine (80 and 8 mg/kg, respectively) anesthesia using aseptic technique as previously described (71–75). Males underwent orchiectomy (scrotal incision), and females underwent ovariectomy (dorsal flank incision), while sham controls received identical procedures without gonad removal. Mice were monitored daily for signs of infection or distress and allowed to recover for 14 days prior to hormone replacement.

### Hormone capsule preparation and implantation

Hormone replacement occurred via implantation of subcutaneous capsules prepared as previously described (71, 72, 76). Silastic tubing (inner diameter 0.04 in, outer diameter 0.085 in) was filled with 5 mm 17β-estradiol (females; Sigma-Aldrich #E8515) or 7 mm testosterone propionate (males; Sigma-Aldrich #T1875), sealed with silicone adhesive (Factor II #A-100), and incubated overnight in 0.9% saline at 37 °C before implantation. Implantation was performed under ketamine-xylazine anesthesia using aseptic surgical technique. Placebo controls received empty capsules and underwent identical surgical procedures.

### Estrogen receptor agonist treatment

Aged females (17 months) received selective estrogen receptor modulators (SERMs) or vehicle via subcutaneous injection every other day from vaccine priming to day 28. Propylpyrazoletriol (PPT; ERα agonist; Tocris #1426) and diarylpropionitrile (DPN; ERβ agonist; Tocris #1494) were reconstituted in DMSO and diluted in medium-chain triglyceride (MCT) oil (60/40 caprylic [C8]/capric [C10]; MedLab #OILSMCTF50ML). Mice received 1 mg/kg of each compound in a 50 μL injection.

### mTOR agonist and antagonist treatment

Adult mice (8–10 weeks) were treated daily with MHY1485 (mTOR agonist; 5 mg/kg; MedChemExpress #HY-B0795) or rapamycin (mTOR antagonist; 8 mg/kg; MedChemExpress #HY-10219) from vaccine priming through 21 dpv. Compounds were reconstituted in DMSO and diluted in phosphate-buffered saline (PBS) before use. Injections were delivered intraperitoneally in 30 μL, with sites alternated daily to reduce irritation. Vehicle controls received DMSO/PBS on the same schedule. Mice were monitored for body mass, rectal temperature, injection-site reactivity, and general distress.

### Statistical analysis

Analyses were performed using GraphPad Prism v10.1.0, R v4.2.3, or MetaboAnalyst 5.0. Comparisons of sex steroid concentrations, antibody and lung viral titers, protein expression, B cell frequencies, intracellular metabolic markers, and reproductive organ weights used unpaired Student’s *t*-tests, one-way or two-way analysis of variance (ANOVA) with Tukey’s *post hoc* test. Body mass changes after viral challenge were analyzed with a repeated-measures ANOVA (mixed-effects model). Flow cytometry data were visualized as R-generated heatmaps (ggplot2) (66) with *Z*-score scaling. All *P*-values were adjusted for multiple comparisons, with adjusted *P* < 0.05 considered significant. Sample sizes and specific tests are provided in figure legends.

For untargeted metabolomics, raw liquid chromatography-mass spectrometry data were log-transformed and normalized to total protein (bicinchoninic acid [BCA] assay) and to sex-matched mock-vaccinated controls. Differential metabolites were defined by unpaired *t*-tests with Benjamini-Hochberg correction (*P* < 0.05, log₂FC > 1, VIP > 1 from OPLS-DA). Volcano plots and OPLS-DA analyses were generated in R (ggplot2) and MetaboAnalyst, and pathway enrichment was performed by Metabolite Set Enrichment Analysis (MSEA) using KEGG annotation (hypergeometric test).

## Data Availability

All relevant data are included in the paper and its supplemental files. No original code was developed in this study. Additional information necessary to reanalyze the data is available from the lead contact upon reasonable request. Requests for information, resources, and reagents should be directed to the lead contact, Sabra L. Klein (sklein2@jhu.edu)

## SUPPLEMENTAL MATERIAL

Supplemental material mbio.03965-25-s0001.docx Supplemental materials and methods and figures.

Table S1 mbio.03965-25-s0002.xlsx Untargeted metabolomics.

Table S2 mbio.03965-25-s0003.xlsx Flow cytometry for metabolic markers in B cell subsets.

ASM does not own the copyrights to Supplemental Material that may be linked to, or accessed through, an article. The authors have granted ASM a non-exclusive, world-wide license to publish the Supplemental Material files. Please contact the corresponding author directly for reuse.
