# Supplementary material for: Estradiol enhances influenza vaccine responses through B cell metabolic reprogramming in female mice
Source: mBio. 2026 Feb 26;17(4):e03965-25. doi: 10.1128/mbio.03965-25 (PMC13059741; doi:10.1128/mbio.03965-25)
Supplement: Supplemental material — s and methods and figures. [file mbio.03965-25-s0001.docx]

**Supplementary Methods and Figures**

**Title: Estradiol Enhances Influenza Vaccine Responses Through B Cell Metabolic Reprogramming in Female Mice**

Authors: Laura A. St. Clair¹, Emily G. Watters¹, Anna Yin¹, Jennifer A. Liu¹, Sabal Chaulagain¹, Elizabeth A. Thompson¹, Sabra L. Klein¹#

Affiliation: ¹W. Harry Feinstone Department of Molecular Microbiology and Immunology, Johns Hopkins University Bloomberg School of Public Health, Baltimore, MD, USA

#Corresponding author:
Sabra L. Klein
Email: [sklein2@jhu.edu](mailto:sklein2@jhu.edu)

**Supplemental Materials and Methods**

*Sample Collection*

Samples were collected 28 days post-vaccination (dpv; 7 days post-boost) or 3 days post-challenge (45 days post-vaccination). Mice were anesthetized with ketamine–xylazine and terminally bled via retro-orbital sinus. At 28 dpv, spleens, draining lymph nodes (inguinal + popliteal, pooled), femoral bone marrow, and blood were collected. Spleens were processed for B-cell isolation (protein and metabolomics analyses) or flow cytometry; lymph nodes and bone marrow for flow cytometry. Tissues were collected in ice-cold FACS buffer (PBS with 2 mM EDTA, 25 mM HEPES, 10 mM sodium fluoride, and 1× Halt Protease/Phosphatase Inhibitor; Corning #46-034-CI, Gibco #15630080, Fisher #S299-100, ThermoFisher #78442). Blood was collected into heparinized tubes, centrifuged at 4°C, and serum separated for antibody (heat-inactivated 56°C for 30 min) and hormone analyses, then stored at −80°C. At 3 days post-challenge, blood and lungs were collected. Lungs were snap-frozen, homogenized in ice-cold DMEM using Lysing Matrix D tubes and the FastPrep-24 system (MP Biomedicals #116913100), centrifuged to remove debris, and supernatants stored at −80°C for viral-titer analysis.

*B Cell Isolation*

Single-cell suspensions were prepared from freshly harvested spleens by gentle mechanical disruption through 70 μm cell strainers (Corning, Cat #352350) using a sterile syringe plunger. Cells were washed twice with cold PBS and then pan–B cells were isolated using the EasySep™ Mouse Pan-B Cell Isolation Kit (STEMCELL Technologies, Cat #19844) according to the manufacturer’s protocol. Isolated B cells were counted and immediately frozen at −80 °C until use in downstream Western blot analysis or metabolomic profiling.

*Antibody measurements*

Antigen-specific antibody titers (IgG, IgG2c, and IgM) were measured using an in-house enzyme-linked immunosorbent assay (ELISA)(4, 5, 7). 96-well plates were coated overnight at 4 °C with 50 μL per well of carbonate–bicarbonate buffer (pH 9.6) containing 2 μg/mL of whole virus antigen. The next day, plates were washed and blocked with 10% milk in PBS for 1 hour at room temperature. Serially diluted plasma samples were added to each well and incubated for 1 hour at room temperature. After washing, horseradish peroxidase (HRP)-conjugated secondary antibodies were added: anti-mouse IgG (ThermoFisher, Cat# 32430, 1:250), anti-mouse IgG2c (STEMCELL Technologies, Cat# 56970, 1:10,000), anti-mouse IgG1 (ThermoFisher, Cat# PA1-74421, 1:10,000), anti-mouse IgG2b (ThermoFisher, Cat# M32507, 1:2000), anti-mouse IgG3 (ThermoFisher, Cat# M32607, 1:2000) or anti-mouse IgM (ThermoFisher, Cat# 62-6820, 1:2,000). Plates were incubated for an additional hour at room temperature, washed, and developed using 3,3′,5,5′-tetramethylbenzidine (TMB) substrate (BD Biosciences, Cat# 555214) for 20 minutes. The reaction was stopped with 1 M hydrochloric acid, and plates were read at 450 nm using a Molecular Devices plate reader. Endpoint titers were defined as the highest dilution with an optical density (OD) greater than the mean OD plus three times the standard deviation of negative control wells.

Neutralizing antibody titers were assessed using a microneutralization assay in Madin-Darby Canine Kidney (MDCK) cells(4, 5, 7). Plasma samples were heat-inactivated at 57 °C for 30 minutes and serially diluted in infection media composed of Dulbecco’s Modified Eagle Medium (DMEM) supplemented with 1% penicillin–streptomycin (ThermoFisher, Cat# 15140122), 1% L-glutamine (ThermoFisher, Cat# A2916801), 0.5% bovine serum albumin (Millipore Sigma,Cat #A9647), and 5 μg/mL N-acetyl trypsin (Millipore Sigma, Cat # T6763). Diluted plasma was incubated with 100 TCID₅₀ of maA/Cal/09 H1N1 virus for 1 hour at room temperature. The virus–plasma mixtures were then added to duplicate wells of confluent MDCK monolayers and incubated for 24 hours at 32 °C. After incubation, the inoculum was removed, cells were washed with PBS, and fresh infection media was added. Plates were incubated at 32 °C for 6 days or until cytopathic effect (CPE) was observed. Cells were fixed with 4% formaldehyde and stained overnight at room temperature with naphthol blue black. Neutralizing titers were defined as the highest dilution of plasma that prevented CPE in 50% of replicate wells.

*Hormone Measurement*

Serum concentrations of E2 and testosterone were measured following acetonitrile extraction. Briefly, two parts serum were mixed with three parts acetonitrile, vortexed, and incubated at room temperature for 10 minutes. Samples were vortexed again and centrifuged at 17,000 × g for 5 minutes at 4°C. The supernatant was transferred to clean tubes and dried in a SpeedVac concentrator (medium heat, maximum speed). Dried extracts were reconstituted in assay buffer provided with each hormone detection kit. E2 concentrations were quantified using the MILLIPLEX® Multi-Species Hormone Magnetic Bead Panel (MilliporeSigma, Cat# MSHMAG-21K) and read on a Luminex INTELLIFLEX system. Testosterone concentrations were measured using the IBL Mouse/Rat Testosterone ELISA Kit (IBL-America, Cat# IB79174) and read on a SpectraMax plate reader. Hormone concentrations were calculated using five-parameter logistic regression curves based on manufacturer-supplied standards.

*Western Blot Analysis*

B cell pellets were lysed in RIPA buffer supplemented with Halt™ protease and phosphatase inhibitors, and protein concentrations were determined using the Pierce™ BCA Protein Assay Kit (ThermoFisher, Cat #23225). Protein expression was assessed using ThermoFisher’s NuPAGE™ system. Samples were prepared in LDS Sample Buffer (Cat #NP0007) with Reducing Agent (Cat #NP0004). To preserve phospho-epitopes, phosphorylated protein samples were not heated; all others were heated at 70 °C for 10 minutes. Equal protein (20 μg) was loaded onto either 4–12% Bis-Tris gels (Cat #NP0322BOX) in MES Buffer (Cat #NP0002) or 3–8% Tris-Acetate gels (Cat #EA0378BOX) in Tris-Acetate buffer (Cat #LA0041), and resolved at 50 V for 30 minutes then 100 V for 1 hour. Proteins were transferred to PVDF membranes using either overnight wet transfer at 30 V (high molecular weight proteins, e.g. mTOR and phospho-mTOR) or the iBlot™ 2 semi-dry transfer system (Cat #IB24001), following manufacturer’s instructions.

Membranes were blocked using Intercept® (TBS) Blocking Buffer (LI-COR, Cat #927-60001), washed with TBS-T, and incubated with primary antibodies overnight at 4 °C. After three washes, membranes were incubated with secondary antibodies for 1–2 hours at room temperature, washed again, and imaged on an Azure 600 imager using fluorescence detection. Band intensities were quantified using ImageJ. Vinculin was selected as a loading control, as its expression has not been linked to mTOR signaling and remained stable across experimental conditions. A complete list of primary and secondary antibodies used for Western blot analysis are listed in **Table S1** at the end of this document.

*Flow Cytometry*

Single-cell suspensions were prepared from spleens, draining lymph nodes (DLNs), and bone marrow by gentle mechanical disruption through 70 μm cell strainers (Corning, Cat #352350) using a sterile syringe plunger. Cells were centrifuged at 700 × g for 5 minutes at 4 °C. Red blood cells were lysed from spleen and bone marrow samples by resuspending cells in 1 mL ACK lysis buffer (Gibco, Cat #118-156-101) for 6 minutes at room temperature. Lysis was performed twice, each time quenched with 1 mL R10 media (RPMI 1640, Corning Cat #15-040-CV; 10% FBS, 1% penicillin/streptomycin, 1% L-glutamine) followed by centrifugation at 700 × g for 5 minutes at 4 °C. Bone marrow and spleen samples were resuspended in 500 μL R10 media, counted, and plated at 1 × 10⁶ cells per well in 96-well plates. DLN cells were resuspended in 250 μL R10 media, and all cells were plated. Plates were centrifuged at 700 × g for 5 minutes at 4 °C to pellet cells. Cells were then washed in 150 μL PBS and centrifuged again at 700 × g for 5 minutes at 4 °C; this wash protocol was used for all subsequent washes.

Cells were stained for viability using Live/Dead Zombie NIR Fixable Viability Dye (BioLegend, Cat #423105) and blocked with TruStain FcX™ PLUS anti-mouse CD16/32 (BioLegend, Cat #156604) for 15 minutes at room temperature. Following a PBS wash, extracellular staining was performed at room temperature in 50 μL of antibody cocktail diluted in 20% BD Horizon™ Brilliant Stain Buffer in PBS for 20 minutes in the dark. Cells were then washed and fixed using the eBioscience™ FoxP3/Transcription Factor Staining Buffer Set (ThermoFisher, Cat #00-5523-00), incubating in Fixation/Permeabilization buffer for 20 minutes at room temperature. After washing with Fix/Perm buffer, intracellular staining was performed in 50 μL of antibody cocktail diluted in Fix/Perm buffer for 20 minutes at room temperature in the dark. Cells were washed sequentially in Fix/Perm buffer and PBS, then resuspended in 150 μL PBS for acquisition. Samples were acquired on a 3-laser Cytek Aurora spectral flow cytometer. FCS files were analyzed using FlowJo v10 (version 10.6.2). All antibodies used for surface and intracellular staining by flow cytometry are detailed in **Table S1** at the end of this document.

*Metabolomics Analysis*

Equal numbers of splenic B cells (2 × 10⁶ per sample) from mock- and maA/Cal09 H1N1-vaccinated mice (n = 10/sex/group, day 28 post-vaccination) were shipped to Novogene Co. Ltd. (Beijing, China) for untargeted metabolomics. Samples were analyzed by LC–MS/MS (ExionLC™ AD system coupled with a SCIEX TripleTOF® 6600+) in both positive and negative ion modes. Metabolites were annotated using Novogene’s in-house library and the HMDB, KEGG, METLIN, and metDNA databases. Only metabolites with an identification score >0.7 and coefficient of variation <0.3 in QC samples were retained. Differential metabolites were defined as those with log₂ fold change >1, adjusted p-value <0.05, and VIP >1.

| **Antibody Name** | **Application** | **Company** | **Catalog Number** | **Fluorophore** | **Host Species** | **Clone** |
| --- | --- | --- | --- | --- | --- | --- |
| Vinculin | Western blot | Bio-Rad | MCA465GA | -- | Mouse | -- |
| CPT1A | Western blot | ThermoFisher | 15184-1-AP | -- | Rabbit | -- |
| Phospho-mTOR (Ser2448) | Western blot | Cell Signaling Technology | 2971S | -- | Rabbit | -- |
| LC3A/B | Western blot | Cell Signaling Technology | 12741S | -- | Rabbit | -- |
| ERα | Western blot | ThermoFisher | PA1-309 | -- | Rabbit | -- |
| ERβ | Western blot | ThermoFisher | PA1-310B | -- | Rabbit | -- |
| Phospho-p70 S6 kinase | Western blot | ThermoFisher | PA5-17671 | -- | Rabbit | -- |
| Goat anti-Rabbit IgG | Western blot (Secondary) | ThermoFisher | A-11008 | Alexa Fluor 488 | Goat | -- |
| Donkey anti-Mouse IgG | Western blot (Secondary) | ThermoFisher | A-31571 | Alexa Fluor 647 | Donkey | -- |
| CD45R/B220 | Flow cytometry | BioLegend | 103237 | BV570 | Rat | RA3-6B2 |
| CD3 | Flow cytometry | BioLegend | 100249 | BV750 | Rat | 17-A2 |
| CD19 | Flow cytometry | BioLegend | 115510 | PE-Cy5 | Rat | 6d5 |
| CD138 | Flow cytometry | BioLegend | 142506 | APC | Rat | 281-2 |
| CD38 | Flow cytometry | BioLegend | 102728 | APC/Cy7 | Rat | 90/CD38 |
| GL7 | Flow cytometry | BioLegend | 144620 | PE/Cy7 | Rat | GL7 RUO |
| CD93 | Flow cytometry | BioLegend | 136504 | PE | Rat | AA4.1 |
| CD21/35 | Flow cytometry | BioLegend | 123416 | PerCP/Cy5.5 | Rat | 7e9 |
| CD23 | Flow cytometry | BioLegend | 101645 | BV785 | Rat | B3B4 |
| PD-L2 | Flow cytometry | BioLegend | 107216 | PE-Dazzle 594 | Rat | TY-25 |
| CD80 | Flow cytometry | BioLegend | 104732 | BV650 | Armenian Hamster | 16-10A1 |
| CD73 | Flow cytometry | BioLegend | 117205 | BV605 | Rat | TY/23 |
| IgD | Flow cytometry | BioLegend | 405723 | BV510 | Rat | 11-26c.2a |
| IgM | Flow cytometry | BioLegend | 406539 | BV711 | Rat | RMM-1 |
| IgG | Flow cytometry | BioLegend | 405334 | PerCP | Goat | poly4053 |
| CD43 | Flow cytometry | BioLegend | 143213 | Alexa Fluor 700 | Rat | S11 |
| GLUT1 | Flow cytometry (intracellular) | Abcam | ab195020 | Alexa Fluor 647 | Rabbit | EPR3915 |
| CPT1A | Flow cytometry (intracellular) | Abcam | ab171449 | Alexa Fluor 488 | Mouse | 8F6AE9 |
| TOMM20 | Flow cytometry (intracellular) | Abcam | ab210047 | Alexa Fluor 405 | Rabbit | EPR15581-54 |
| Phospho-S6 | Flow cytometry (intracellular) | Cell Signaling Technology | 8520 | Pacific Blue | Rabbit | D57.2.2E |

**Table S1 - Antibodies used in Western blot and flow cytometry experiments.**


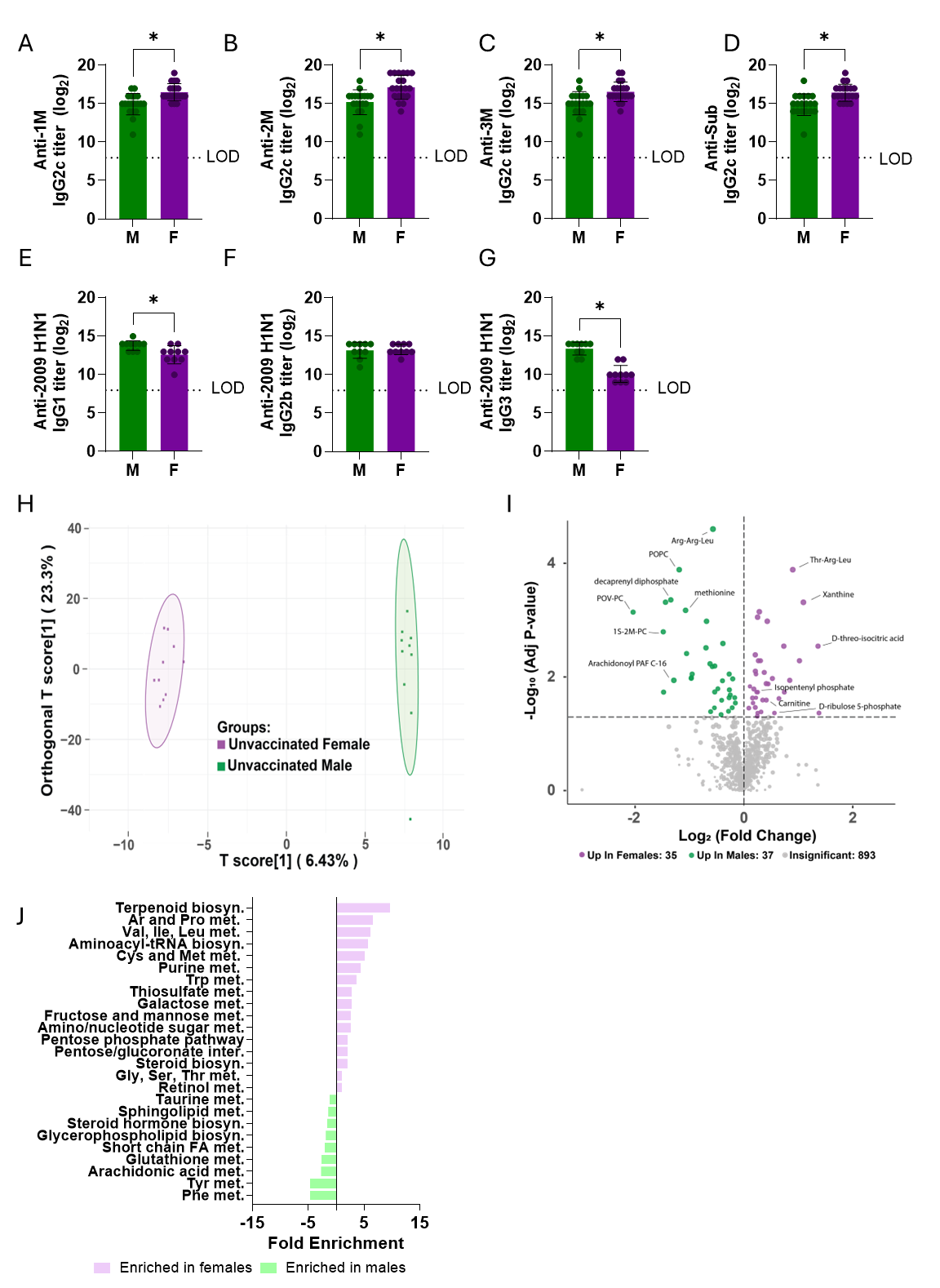


**Supplemental Figure 1**

**Supplemental Figure 1. Females generate broader antibody responses against H1N1 drift variants and exhibit distinct baseline metabolic profiles**. (A–G) Serum collected 28 dpv from vaccinated male (green) and female (purple) mice was assessed for IgG2c titers by ELISA against maA/Cal/09 variants, including (A) 1M (single point mutation), (B) 2M (two point mutations), (C) 3M (three point mutations), and (D) Sub (one point mutation with Ca2 epitope substituted with an H5-derived sequence) as well as additional isotypes including (E) IgG1, (F) IgG2b, and (G) IgG3. Dashed lines indicate the limit of detection (LOD). (H) OPLS-DA plot showing separation of splenic metabolite profiles between mock-vaccinated males and females. (I) Volcano plot of all identified metabolites in unvaccinated animals. Purple and green dots represent metabolites significantly enriched in females and males, respectively. Labeled points highlight metabolites of interest. (J) Quantitative Metabolite Set Enrichment Analysis (QSEA) of significantly altered metabolites, performed using MetaboAnalyst 5.0 with KEGG pathway annotation. Enrichment p-values were calculated using a hypergeometric test. Statistical analyses: (A-G) Student’s t-test; (H) OPLS-DA model with permutation testing (200 iterations); (I) FDR-adjusted p-values (p < 0.05) were calculated using unpaired two-sided t-tests with Benjamini–Hochberg correction (J) Hypergeometric test for pathway enrichment. *p < 0.05.

**Supplemental Figure 2**

**
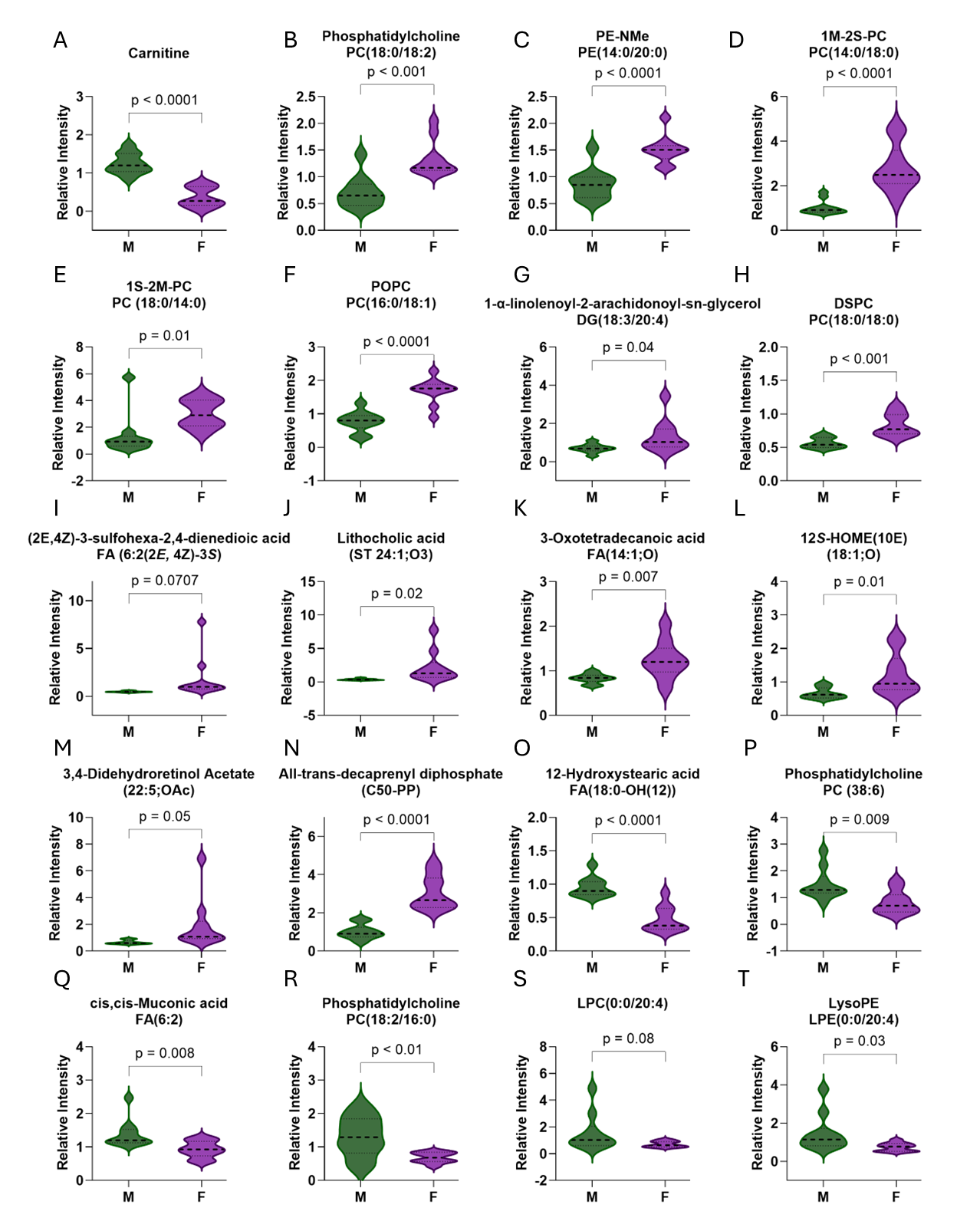
**

**Supplemental Figure 2.** Relative abundance of differentially expressed lipid metabolites identified in splenic B cells from vaccinated male and female mice. Violin plots show individual data points and median (dashed line) from samples collected 28 dpv. Adjusted p-values calculated using the two-stage step-up method of Benjamini, Krieger, and Yekutieli. Values represent metabolite intensities normalized to same-sex mock controls.

**
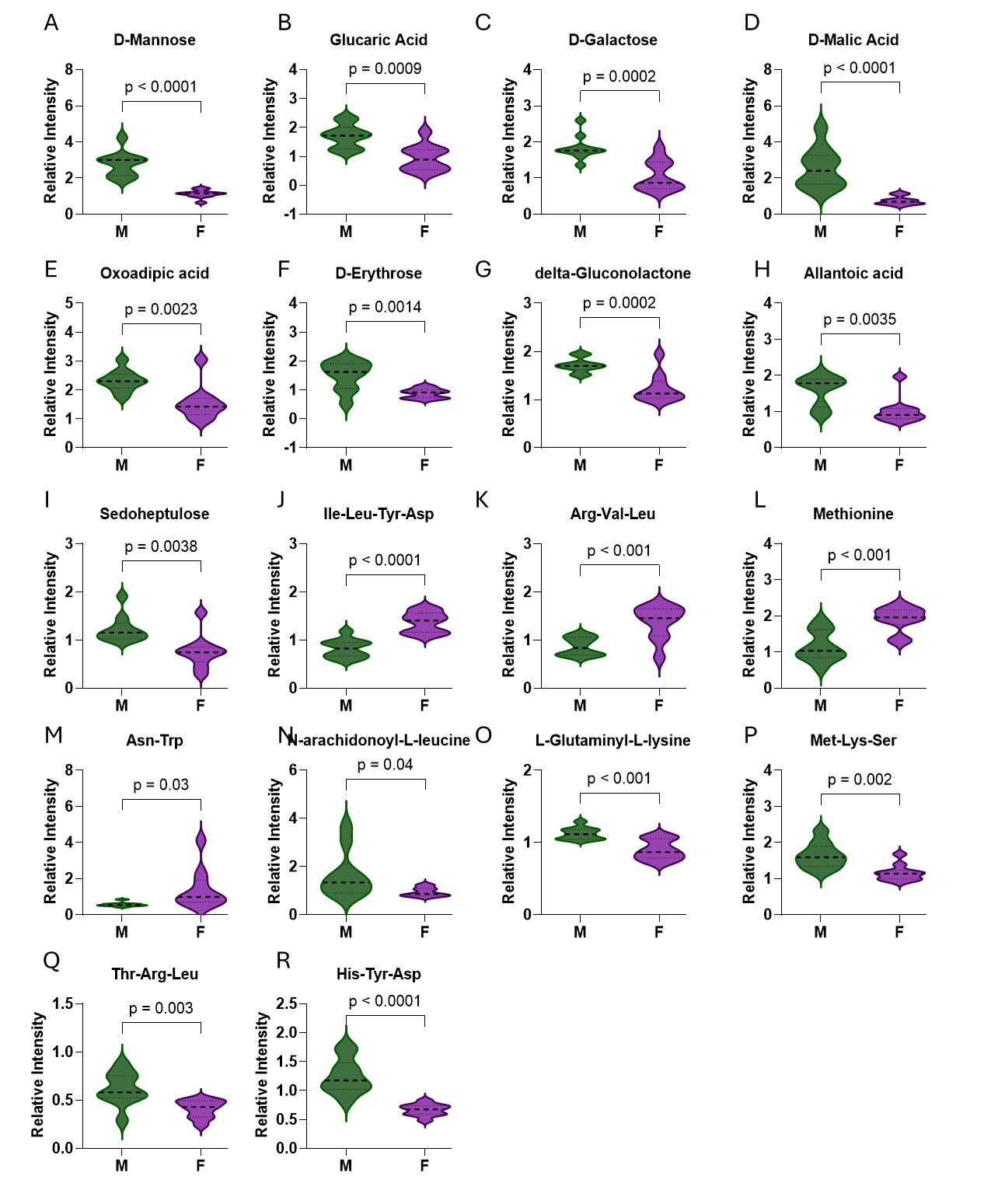
Supplemental Figure 3**

**Supplemental Figure 3. Relative abundance of top differentially expressed central carbon and amino acid metabolites in splenic B cells from vaccinated male and female mice**. Violin plots show individual data points and median (dashed line) from samples collected 28 dpv. Adjusted p-values calculated using the two-stage step-up method of Benjamini, Krieger, and Yekutieli. Values represent metabolite intensities normalized to same-sex mock controls.

**Supplemental Figure 4**

**
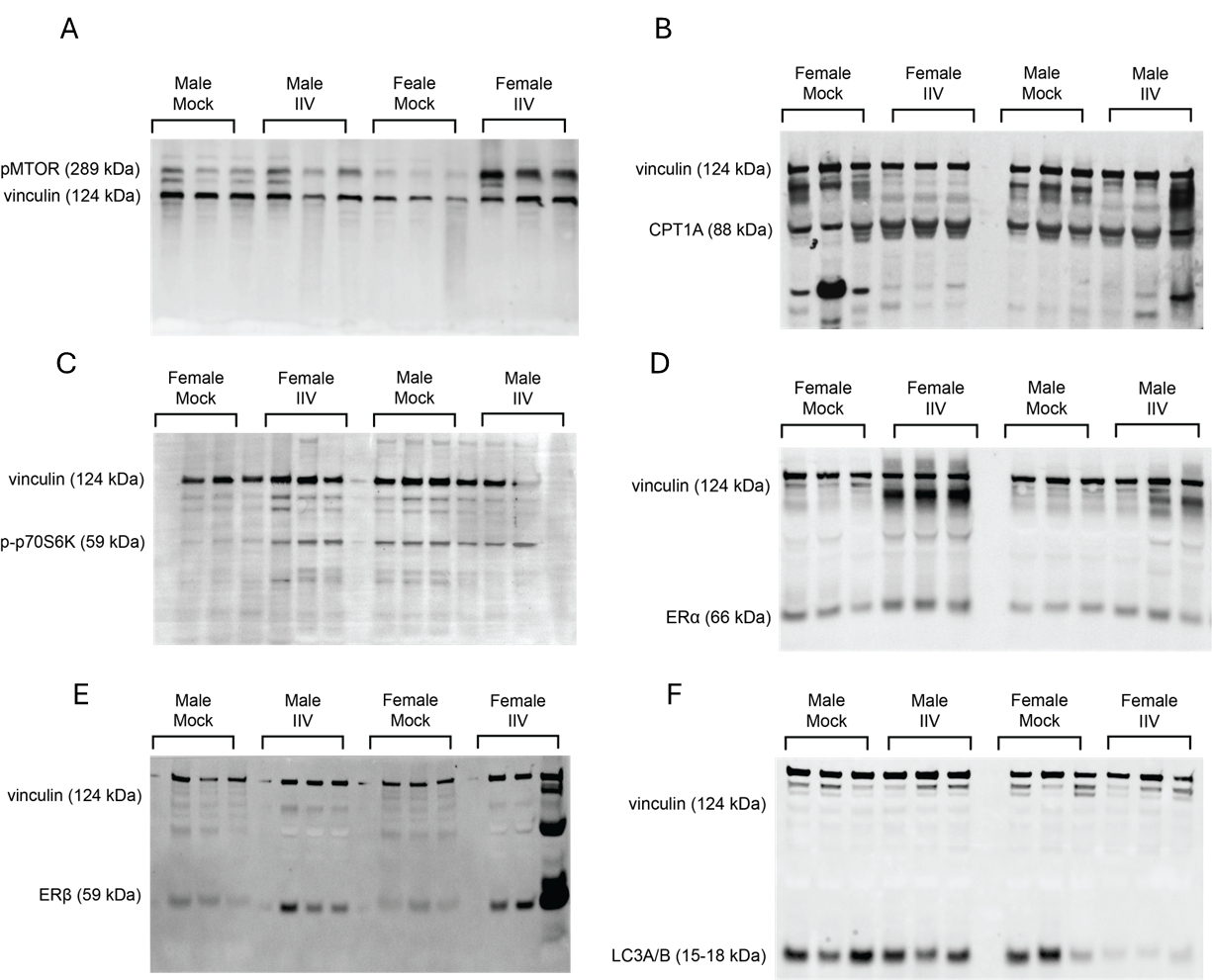
**

**Supplemental Figure 4. Original Western blot images corresponding with Fig 3.** Adult male and female mice were vaccinated and boosted as in Fig. 1 (n=3/sex/group). At 28 days post-vaccination, splenic B cells were isolated, and whole-cell protein lysates were analyzed by Western blot. (A-F) Western blot images of phosphorylated mTOR (p-mTOR) (A), CPT1a (B), phosphorylated p70 S6 kinase (p-p70S6K) (C), estrogen receptor alpha (ERα) (D), estrogen receptor beta (ERβ) (E), and LC3A/B (F) in splenic B cells. Images were captured using Azure 600 Imager.

**Supplemental Figure 5**

**
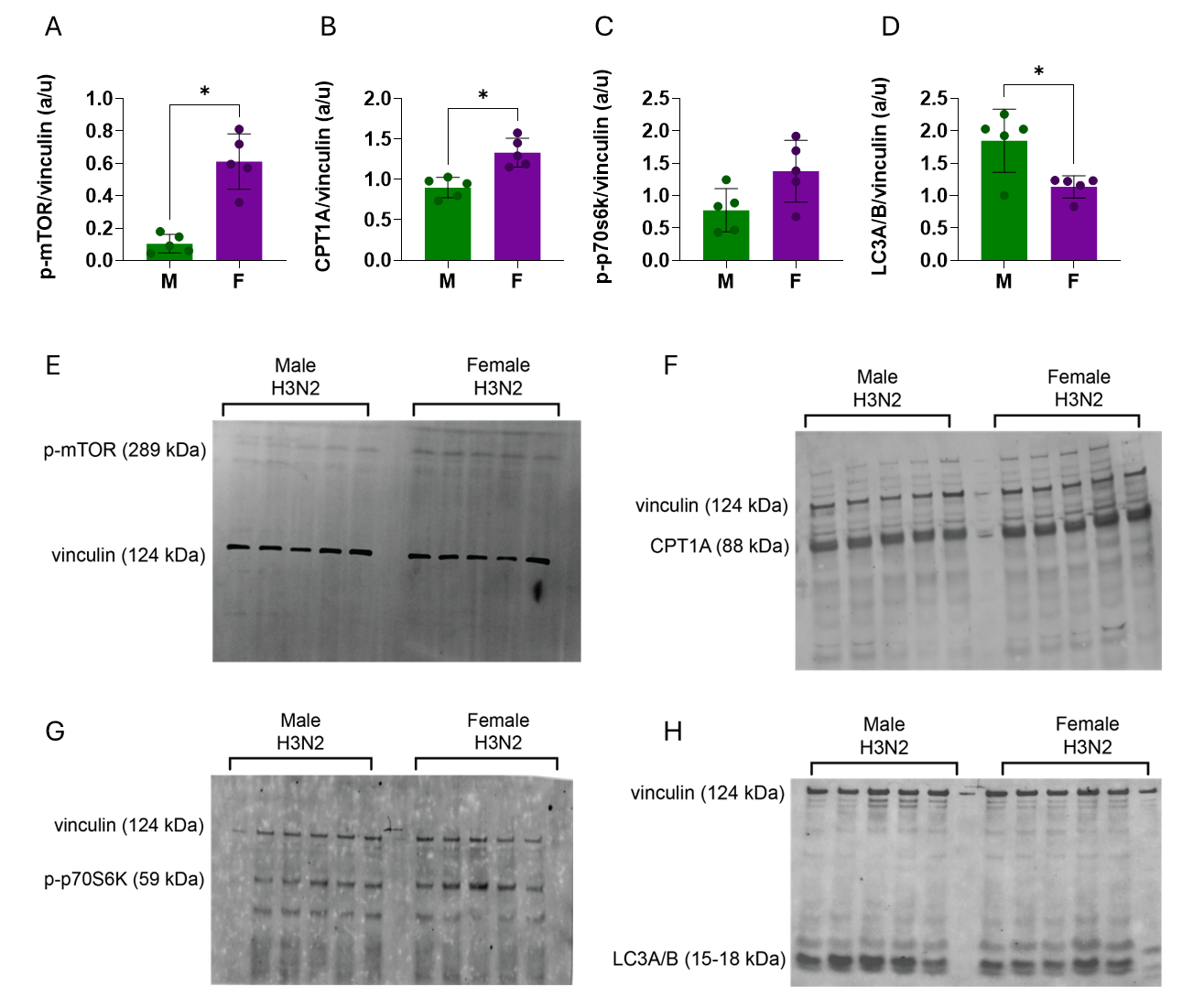
**

**Supplemental Figure 5. Sex differential mTOR signaling following H3N2 vaccination.** Adult male and female mice were vaccinated and boosted with an inactivated maA/HK/68/H3N2 vaccine as in Fig. 1 (n=5/sex). At 28 days post vaccination, splenic B cells were isolated, and whole-cell protein lysates were analyzed by Western blot. (A-D) Densitometric quantification of p-mTOR (E), CPT1a (F), p70 S6K (G), and LC3A/B (H), were normalized to vinculin (loading control). (E-H) Western blot images of p-mTOR (E), CPT1A (F), p-p70S6K (G), and LC3A/B (H) in splenic B cells. Images were captured using Azure 600 Imager, and values were quantified using ImageJ. Bar graphs display individual data points with mean ± SEM. Statistical comparisons were performed using unpaired two-tailed Student’s t-tests, * p < 0.05.

**Supplemental Figure 6**

**
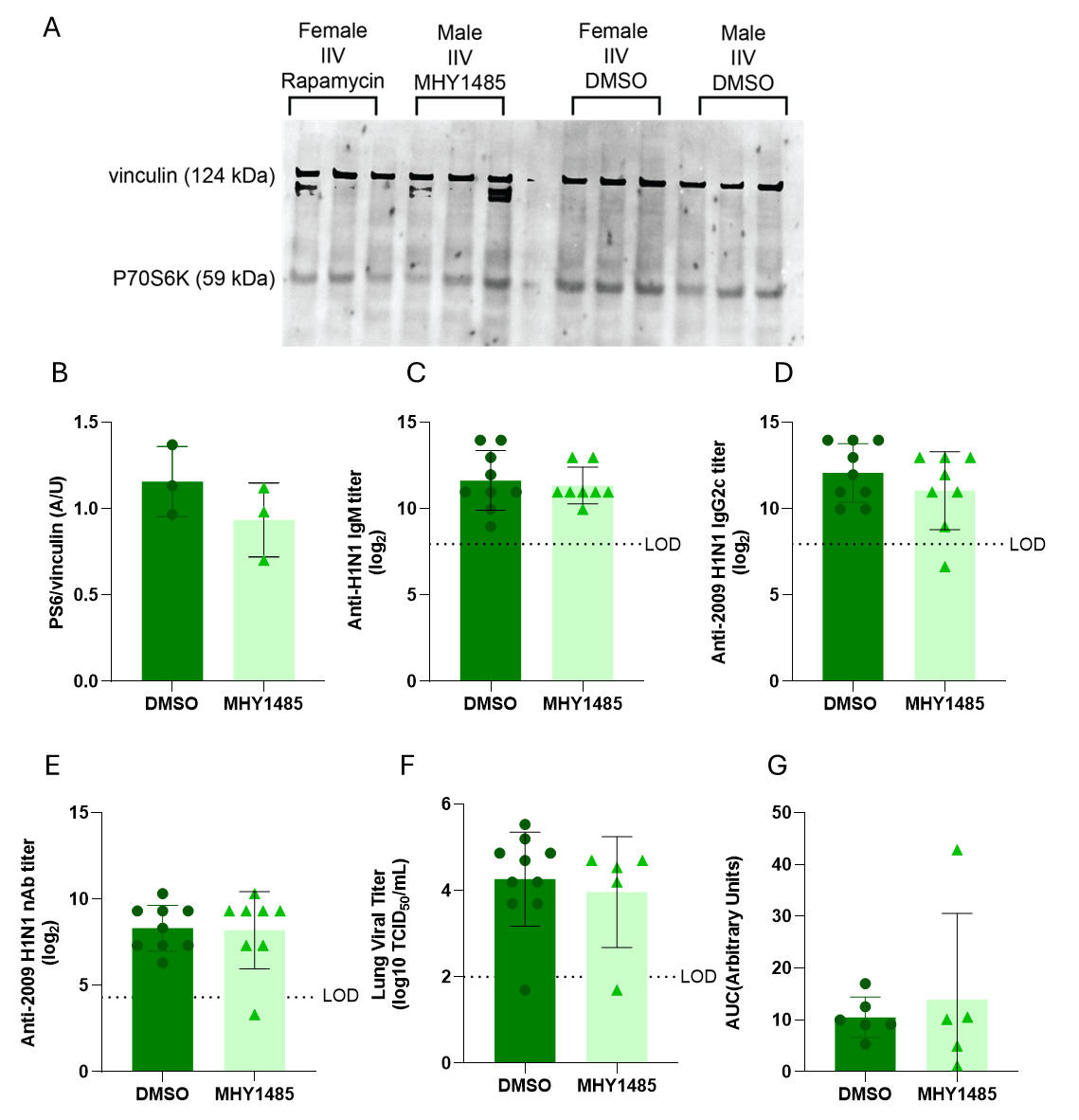
**

**Supplemental Figure 6. mTOR agonist treatment does not affect mTOR signaling in B cells from male mice.** Adult male mice were treated daily by i.p. injection with either vehicle control or 5 mg/kg MHY1485 for 21 days and were vaccinated and boosted as described in Fig 1 (n=10/treatment). At 28 days post vaccination (dpv), a subset of mice (n=3/treatment) were euthanized to measure protein expression of p-p70S6K (A-B). (A) Original Western blot image of p-p70S6K in splenic B cells from vaccinated, antagonist-treated females and agonist-treated males. Image captured on Azure 600 Imager, and (B) Densiometric analysis of p-p70S6K using ImageJ. All remaining mice were also bled at 28 dpv for analysis of anti-H1N1 IgM (C), anti-H1N1 IgG2c (D), and nAb (E) titers. Mice were challenged at 42 dpv, and morbidity was assessed via analysis of lung viral titers at 3 DPC (F), and change in body mass which is displayed as absolute area under the curve (AUC; G). Each point represents an individual mouse. Bars indicate mean ± SEM. Statistical significance was determined by student’s T test, *p<0.05.

**
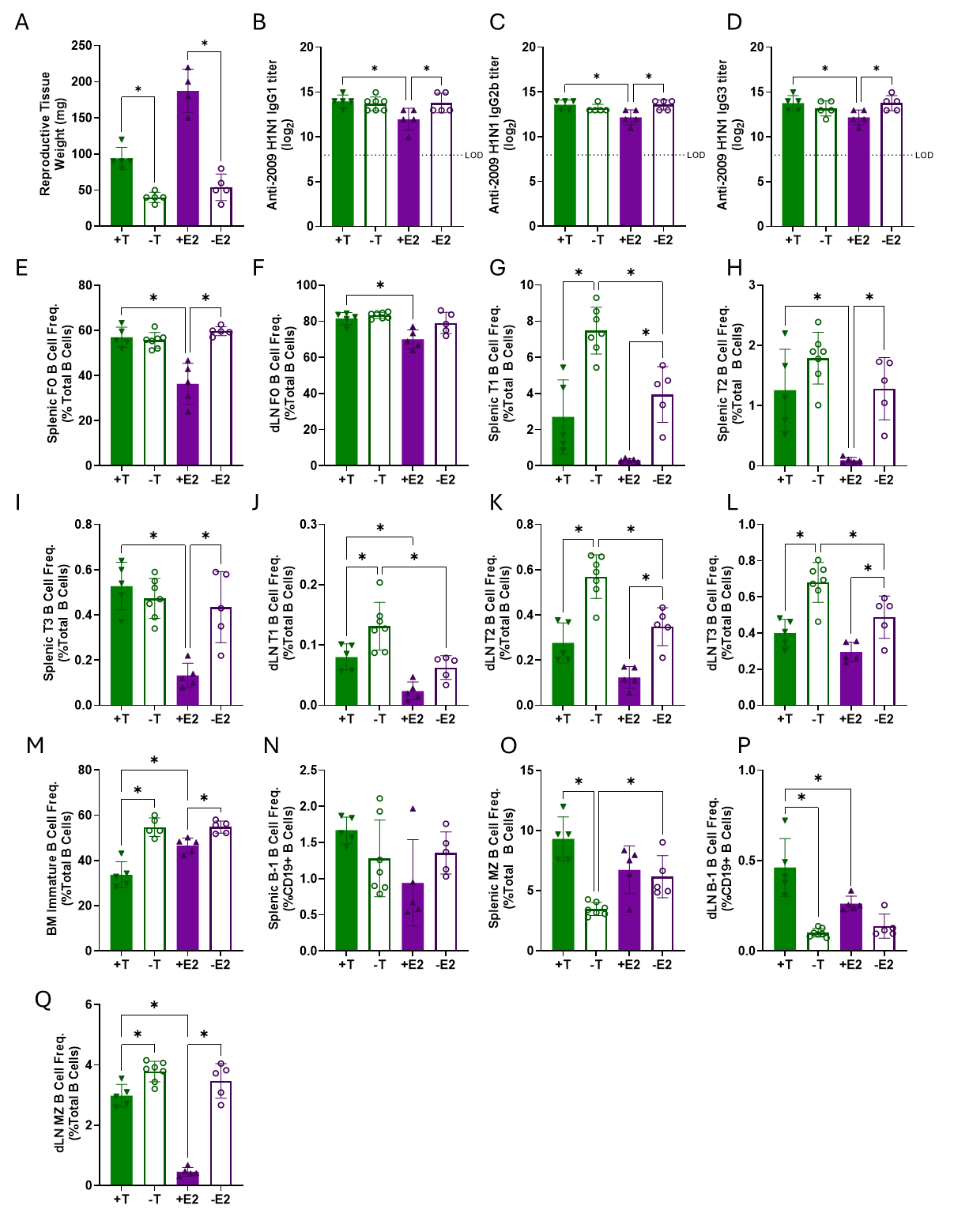
Supplemental Figure 7**

**Supplemental Figure 7. Frequencies of B cell subsets following hormone manipulation and vaccination.** (A) Reproductive tissue weight from gonadectomized, hormone treated mice. (B-D) IgG1, IgG2b, and IgG3 antibody titers from serum collected at 28 dpv. Frequencies of splenic and dLN FO B cells (E-F), splenic and dLN T1-3 B cells (G-L), immature bone marrow B cells (M), and splenic and dLN innate-like B cells (B1-B and MZ B cells, N-Q). Statistical analysis: one-way ANOVA with Tukey’s multiple comparisons, *p < 0.05.

**
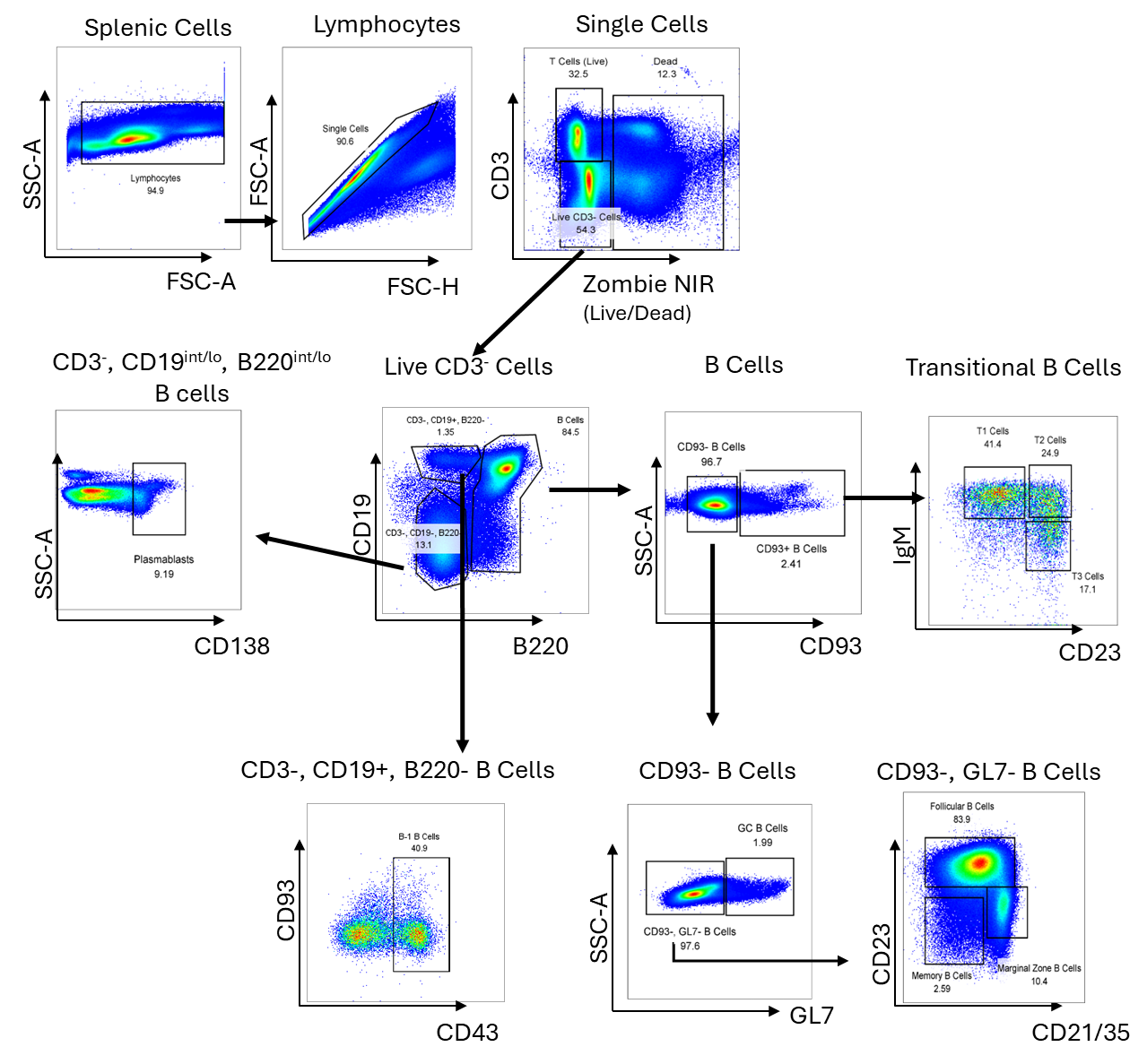
Supplemental Figure 8**

**Supplemental Figure 8. Flow cytometry gating strategy for identification of B cell subsets.** Representative plots showing the sequential gating used to define splenic, draining lymph node (dLN), and bone marrow B cell populations. Subsets include transitional (T1, T2, T3), follicular (FO), marginal zone (MZ), germinal center (GC), plasmablasts, plasma cells, and B-1 B cells. Gates were set based on fluorescence minus one (FMO) controls and biological distribution of populations.

**
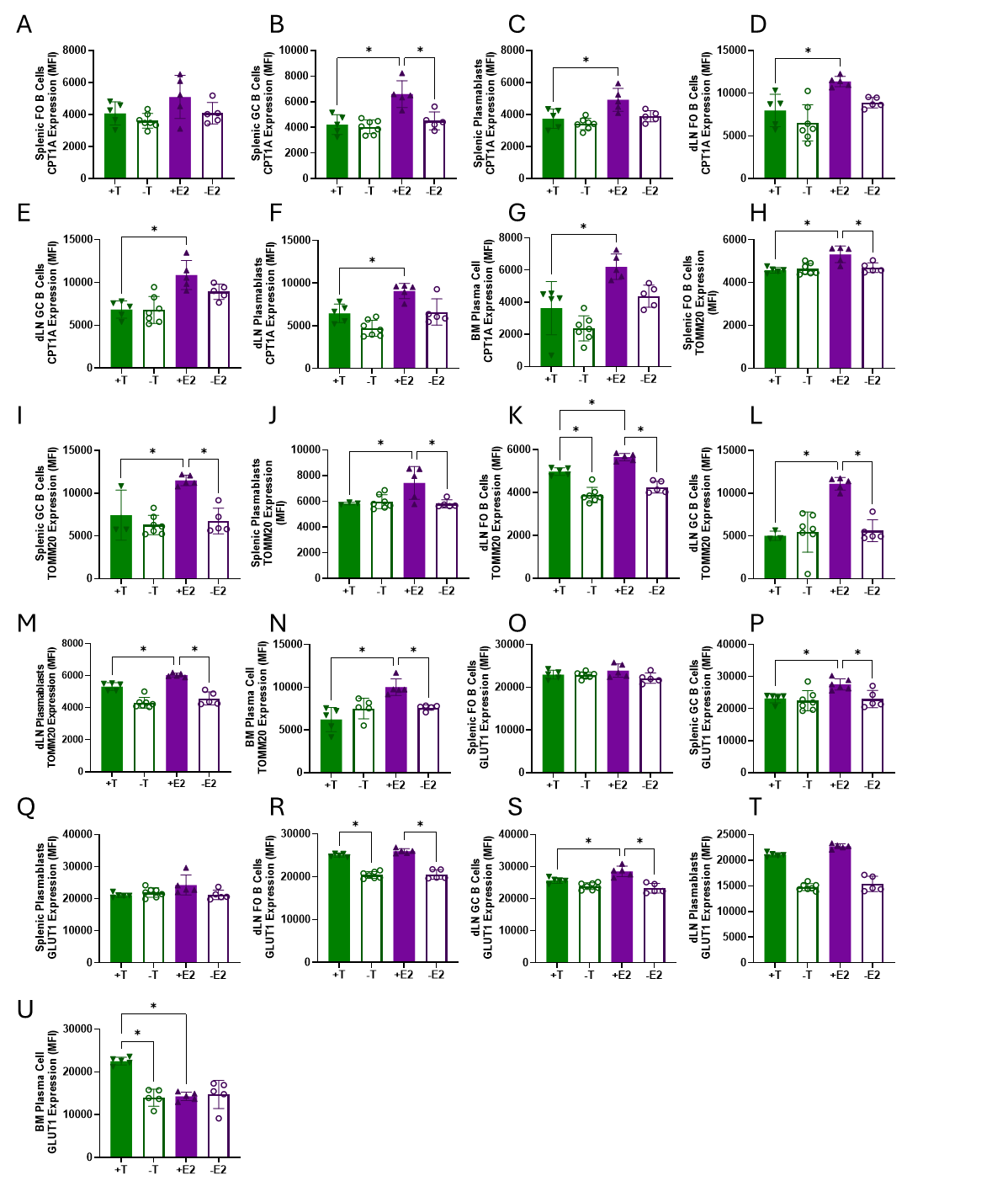
Supplemental Figure 9**

**Supplemental Figure 9. Expression of intracellular metabolic proteins in antibody-secreting B cell subsets.** CPT1A (A-G), TOMM20 (H-N), and GLUT1 (O-U) expression in splenic and dLN FO, GC, and plasmablasts and BM plasma cells. Statistical analysis: one-way ANOVA with Tukey’s multiple comparisons, *p < 0.05.


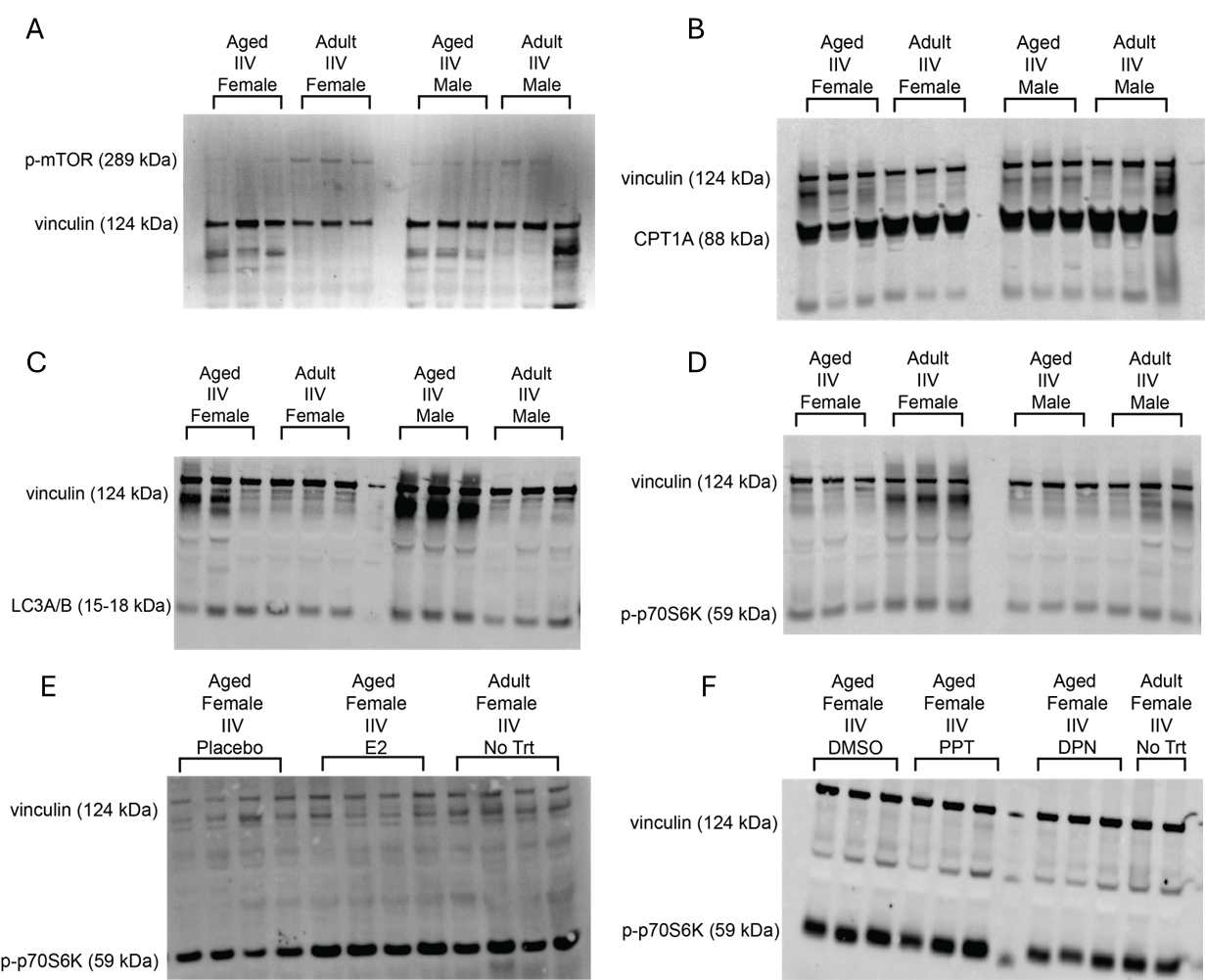
**Supplemental Figure 10**

**Supplemental Figure 10. Original Western blot images for mTOR-pathway proteins shown in Figure 7.** (A–D) Western blot membranes showing expression of p-mTOR (A), CPT1a (B), p-p70S6K (C), and LC3A/B (D) in total splenic B cells from adult and aged vaccinated male and female mice. (E) Western blot for p-p70S6K expression in splenic B cells from adult, aged placebo-treated, and aged E2-treated vaccinated female mice. (F) Western blot for p-p70S6K expression in splenic B cells from aged vaccinated females treated with vehicle (DMSO), PPT (ERα agonist), or DPN (ERβ agonist). Images obtained using Azure 600 Imager.
